# A Statewide Quality Initiative to Reduce Unnecessary Antibiotic Treatment of Asymptomatic Bacteriuria

**DOI:** 10.1001/jamainternmed.2023.2749

**Published:** 2023-07-10

**Authors:** Valerie M. Vaughn, Ashwin Gupta, Lindsay A. Petty, Anurag N. Malani, Danielle Osterholzer, Payal K. Patel, Mariam Younas, Steven J. Bernstein, Stephanie Burdick, David Ratz, Julia E. Szymczak, Elizabeth McLaughlin, Tawny Czilok, Tanima Basu, Jennifer K. Horowitz, Scott A. Flanders, Tejal N. Gandhi

**Affiliations:** 1 Division of General Internal Medicine, Department of Internal Medicine, University of Utah School of Medicine, Salt Lake City; 2 Division of Health System Innovation & Research, Department of Population Health Sciences, University of Utah School of Medicine, Salt Lake City; 3 Division of Hospital Medicine, Department of Internal Medicine, Michigan Medicine, University of Michigan, Ann Arbor; 4 Medicine Service, Veterans Affairs Ann Arbor Healthcare System, Ann Arbor, Michigan; 5 Division of Infectious Diseases, Department of Internal Medicine, Michigan Medicine, University of Michigan, Ann Arbor; 6 Division of Infectious Diseases, Department of Internal Medicine, Trinity Health, St Joseph Mercy Ann Arbor, Ann Arbor, Michigan; 7 Division of Infectious Diseases, Hurley Medical Center, Flint, Michigan; 8 Division of Infectious Diseases and Clinical Epidemiology, Intermountain Healthcare, Salt Lake City, Utah; 9 Division of General Internal Medicine, Department of Internal Medicine, Michigan Medicine, University of Michigan, Ann Arbor; 10 Division of Hospital Medicine, Corewell Health, Grand Rapids, Michigan; 11 Center for Clinical Management Research, Veterans Affairs Ann Arbor Healthcare System, Ann Arbor, Michigan; 12 Division of Epidemiology, Department of Internal Medicine, University of Utah School of Medicine, Salt Lake City; 13 Division of Infectious Diseases, Department of Medicine, Perelman School of Medicine, University of Pennsylvania, Philadelphia; 14 Department of Internal Medicine, College of Human Medicine, Michigan State University, East Lansing

## Abstract

**Importance:**

Hospitalized patients with asymptomatic bacteriuria (ASB) often receive unnecessary antibiotic treatment, which increases antibiotic resistance and adverse events.

**Objective:**

To determine whether diagnostic stewardship (avoiding unnecessary urine cultures) or antibiotic stewardship (reducing unnecessary antibiotic treatment after an unnecessary culture) is associated with better outcomes in reducing antibiotic use for ASB.

**Design, Setting, and Participants:**

This 3-year, prospective quality improvement study included hospitalized general care medicine patients with a positive urine culture among 46 hospitals participating in a collaborative quality initiative, the Michigan Hospital Medicine Safety Consortium. Data were collected from July 1, 2017, through March 31, 2020, and analyzed from February to October 2022.

**Exposure:**

Participation in the Michigan Hospital Medicine Safety Consortium with antibiotic and diagnostic stewardship strategies at hospital discretion.

**Main Outcomes and Measures:**

Overall improvement in ASB-related antibiotic use was estimated as change in percentage of patients treated with antibiotics who had ASB. Effect of diagnostic stewardship was estimated as change in percentage of patients with a positive urine culture who had ASB. Effect of antibiotic stewardship was estimated as change in percentage of patients with ASB who received antibiotics and antibiotic duration.

**Results:**

Of the 14 572 patients with a positive urine culture included in the study (median [IQR] age, 75.8 [64.2-85.1] years; 70.5% female); 28.4% (n = 4134) had ASB, of whom 76.8% (n = 3175) received antibiotics. Over the study period, the percentage of patients treated with antibiotics who had ASB (overall ASB-related antibiotic use) declined from 29.1% (95% CI, 26.2%-32.2%) to 17.1% (95% CI, 14.3%-20.2%) (adjusted odds ratio [aOR], 0.94 per quarter; 95% CI, 0.92-0.96). The percentage of patients with a positive urine culture who had ASB (diagnostic stewardship metric) declined from 34.1% (95% CI, 31.0%-37.3%) to 22.5% (95% CI, 19.7%-25.6%) (aOR, 0.95 per quarter; 95% CI, 0.93-0.97). The percentage of patients with ASB who received antibiotics (antibiotic stewardship metric) remained stable, from 82.0% (95% CI, 77.7%-85.6%) to 76.3% (95% CI, 68.5%-82.6%) (aOR, 0.97 per quarter; 95% CI, 0.94-1.01), as did adjusted mean antibiotic duration, from 6.38 (95% CI, 6.00-6.78) days to 5.93 (95% CI, 5.54-6.35) days (adjusted incidence rate ratio, 0.99 per quarter; 95% CI, 0.99-1.00).

**Conclusions and Relevance:**

This quality improvement study showed that over 3 years, ASB-related antibiotic use decreased and was associated with a decline in unnecessary urine cultures. Hospitals should prioritize reducing unnecessary urine cultures (ie, diagnostic stewardship) to reduce antibiotic treatment related to ASB.

## Key Points

**Question:**

Is stewardship focused on diagnostic testing or antibiotic treatment associated with reduced asymptomatic bacteriuria (ASB) treatment in hospitalized patients?

**Findings:**

In this 3-year, 46-hospital quality improvement study including 14 572 patients with positive urine cultures, the percentage of patients with a positive urine culture who had ASB declined from 34.1% to 22.5%, suggesting more selective urine testing. In contrast, the percentage of patients with ASB who received antibiotics and antibiotic duration for ASB remained relatively stable.

**Meaning:**

To decrease antibiotic treatment related to ASB, hospitals should prioritize reducing unnecessary urine cultures (ie, diagnostic stewardship).

## Teaser

This quality improvement study aims to determine whether diagnostic stewardship (avoiding unnecessary urine cultures) or antibiotic stewardship (reducing unnecessary antibiotic treatment after an unnecessary culture) is associated with better outcomes in reducing antibiotic use for asymptomatic bacteriuria.

## Introduction

Hospitalized patients are at high risk of having asymptomatic bacteriuria (ASB).^1,2,3,4^ With the exception of certain populations (eg, pregnant individuals), treatment of ASB with antibiotics does not improve outcomes.^5,6,7^ Rather, treatment exposes patients to antibiotic-associated harm, including adverse drug events and antibiotic resistance, and for hospitalized patients may increase length of stay.^8,9,10^

Despite national guidelines recommending against treating ASB in most hospitalized patients,^5^ up to 80% are treated with antibiotics.^8,11,12^ To combat this antibiotic overuse, both diagnostic stewardship (avoiding unnecessary urine cultures) and antibiotic stewardship (avoiding or stopping unnecessary antibiotic use in asymptomatic patients found to have a positive urine culture) have been proposed.^13^ Diagnostic stewardship uses the underlying principle that a positive culture—even in an asymptomatic patient—is a powerful stimulus for treatment and leads to a knee-jerk reaction to prescribe antibiotics.^14^ Thus, the ultimate goal is to improve culturing practices. Successful urine culture stewardship strategies include reflex urine cultures, order-set hygiene, suppressing urine culture results, or education.^15,16,17,18,19,20,21^ Although these efforts have reduced ASB treatment in single-center studies, data from large collaboratives are lacking. In addition, it is unclear whether efforts to reduce ASB treatment should focus on diagnostic or antibiotic stewardship.

In 2017, the Michigan Hospital Medicine Safety Consortium (HMS), a statewide collaborative quality initiative, began collecting data, sharing best practices, and benchmarking performance related to antibiotic treatment of ASB. In this quality improvement study, we report the outcomes of the collaborative and estimate the effect of diagnostic vs antibiotic stewardship on ASB treatment.

## Methods

### Study Design

Descriptions of HMS have been published previously.^8,22,23,24,25,26^ Briefly, HMS is a statewide collaborative quality initiative sponsored by Blue Cross Blue Shield of Michigan and Blue Care Network to improve care for hospitalized medical patients. Of the 92 hospitals in Michigan, 50 (54.3%) participated during the study period. We report through March 31, 2020 (around the beginning of the COVID-19 pandemic).

In 2017, HMS began collecting data on hospitalized medical patients treated for urinary tract infection (UTI; see eFigure 1 in Supplement 1 for timeline). To improve patient care, HMS uses 3 quality improvement pillars: (1) data with benchmarking, (2) sharing of best practices, and (3) pay-for-performance data.^24^ First, HMS collects patient-level data from each hospital through medical record review by nurse abstractors employed by individual hospitals but trained and supported by HMS. Patient data include signs and symptoms, diagnostic testing, antibiotic prescriptions, and outcomes.^8^ These data inform hospital quality metrics that are shared to benchmark performance and identify areas for improvement^27^ (example reports are available in the eAppendix in Supplement 1). Second, during triannual meetings and via an online toolkit,^28^ HMS disseminates best practices related to UTI care, including diagnostic and antibiotic stewardship strategies. These meetings include successful strategies from top hospitals and brainstorming sessions to engage struggling hospitals (see examples in the eAppendix in Supplement 1). How hospitals use HMS resources is left to their discretion but is assessed through an annual survey. Third, HMS selects certain metrics to become pay-for-performance metrics that, if met, provide additional funding for hospitals. Since 2018, 1 HMS pay-for-performance metric has targeted diagnostic and antibiotic stewardship related to ASB (details in the eAppendix in Supplement 1).

Because the purpose of HMS is to measure and improve the quality of existing care practices, this project was determined by the University of Michigan’s institutional review board to be nonregulated, as it did not satisfy the definition of human participant research. We followed Standards for Quality Improvement Reporting Excellence (SQUIRE) 2.0 reporting guidelines.

### Patient Population

Patients were eligible for inclusion if they were hospitalized at an HMS hospital, had a positive urine culture, and did not receive antibiotic treatment for another infection during hospitalization. Patients ineligible for inclusion were those with any of the following: concomitant infection (eg, cellulitis; defined by medical record review), initial admission to intensive care, pregnancy, severe immune compromise (eg, transplant, neutropenia, HIV with CD4 count <200 cells/mm^3^), or urine culture positive only for candida. Patients were also ineligible if they had altered urinary tract anatomy or a urologic procedure during hospitalization or were receiving antibiotic treatment for a UTI at the time of admission. Patients with symptoms of a UTI who did not receive antibiotics were excluded.

Patients were considered to have ASB if they had no signs or documented symptoms of a UTI based on national guidelines.^5,8,27^ Briefly, patients were considered to have ASB if they did not have documentation of any of the following: urgency, frequency, dysuria, suprapubic/costovertebral pain/tenderness, acute hematuria, fever (>38 °C), rigors, or pyelonephritis. Patients with new-onset mental status changes without other symptoms of a UTI were considered to have a UTI only if they had leukocytosis (>10 000 cells/uL), hypotension (<90 mm Hg), or at least 2 systemic inflammatory response syndrome (SIRS) criteria. Patients with bacteremia from a urinary source or severe sepsis (≥2 SIRS criteria plus organ damage) were considered to have a UTI. Patients were considered treated for a UTI if they received antibiotic therapy within 3 days of the positive urine culture. The goal for each hospital was to include 8 patients every 16 days, including weekends. To identify patients for inclusion, HMS abstractors consecutively screened positive urine culture lists in order of discharge time. Once 1 case was included (or if no eligible cases were identified that day), the abstractor would screen the subsequent discharge day until 8 patients were included. Abstractors undergo case-based training and random yearly audits by HMS for quality assurance (details in the eAppendix in Supplement 1).^26,29^ Hospital characteristics were collected from self-report or publicly available databases (details in the eAppendix in Supplement 1). Antibiotic and diagnostic stewardship strategies were collected from self-report via 2019 or 2018 hospital surveys (details in the eAppendix in Supplement 1).

### Primary Outcomes

**Figure 1.**
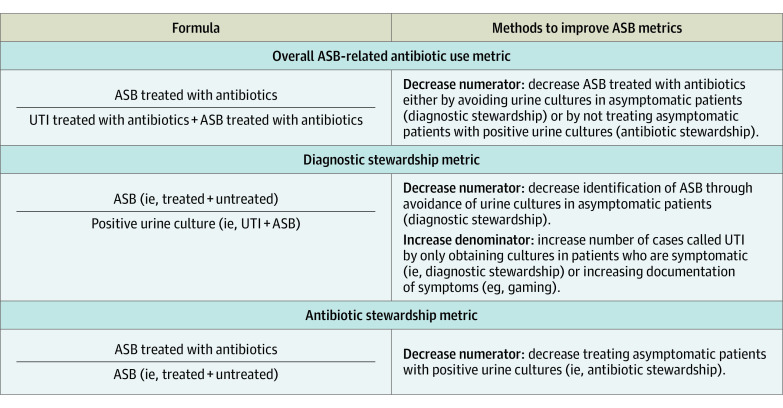
Asymptomatic Bacteriuria (ASB) Metrics The 3 ASB metrics and their association with antibiotic and diagnostic stewardship are shown. Notably, because there is a fixed number of urine culture samples over time, if the number of ASB cases decreases, the sample will be enriched with urinary tract infection (UTI) cases and vice versa.

The association between the primary outcomes and how each could be improved through diagnostic vs antibiotic stewardship is shown in Figure 1. Overall ASB-related antibiotic use was assessed using a metric endorsed by the National Quality Forum for measuring inappropriate diagnosis of UTI: the percentage of patients treated for a UTI who actually have ASB.^23,27^ This metric was used because it measures improvement that happens both due to diagnostic stewardship (ie, more selective urine culturing) and antibiotic stewardship (ie, avoiding antibiotic treatment in patients in whom ASB has been identified).

#### Diagnostic Stewardship

Because we only sampled patients with a positive urine culture, patients who had ASB did not enter the cohort when a urine culture was not collected. If diagnostic stewardship was associated with positive outcomes, the percentage of positive urine cultures that were ASB would decrease over time; thus, the diagnostic stewardship metric was the percentage of positive urine cultures that were ASB, regardless of antibiotic treatment.

#### Antibiotic Stewardship

We measured the estimated effect of antibiotic stewardship in 2 ways. First, we characterized the change in the percentage of patients identified to have ASB who received antibiotic therapy. Second, we characterized the change in antibiotic duration in patients with ASB. Additional outcomes of interest included estimated ASB cases and antibiotic treatment days avoided, hospital characteristics associated with improvements in antibiotic use over time, and self-reported diagnostic and antibiotic stewardship strategies conducted by participating HMS hospitals.

### Sensitivity Analyses

The HMS consortium does not continuously collect rates of urine cultures over time. To confirm changes in urine culture practices over time, HMS conducted 3 separate, 2 week–long prevalence surveys of urine cultures. During the 3 time periods (June 14-27, 2018, April 4-17, 2019, and April 28 to May 11, 2022), hospitals reviewed all urine cultures and reported the total number of cases that met HMS inclusion criteria.

As noted in Figure 1, ASB treatment—particularly the diagnostic stewardship metric—could appear to improve if documentation changed over time (eg, improved/more documentation of urinary symptoms). If this were the case, we would expect the percentage of patients classified as having a UTI who have objective signs of a UTI (which do not rely on documentation, such as leukocytosis) to decrease over time while subjective findings (eg, dysuria) increase as documentation increases. To evaluate for possible documentation changes, we assessed for change over time in objective signs (fever, hypotension, leukocytosis, SIRS criteria) vs subjective symptoms assessed via documentation (urgency, rigors, frequency, dysuria, suprapubic pain/tenderness, acute hematuria, costovertebral/flank pain/tenderness, documentation of pyelonephritis, altered mental status) in patients classified as having a UTI.

### Statistical Analysis

We first described the percentage of patients with a positive urine culture who had ASB (treated vs not) and UTI, as well as the change over time in each group. We then characterized self-reported diagnostic or antibiotic stewardship strategies using descriptive statistics.

**Figure 2.**
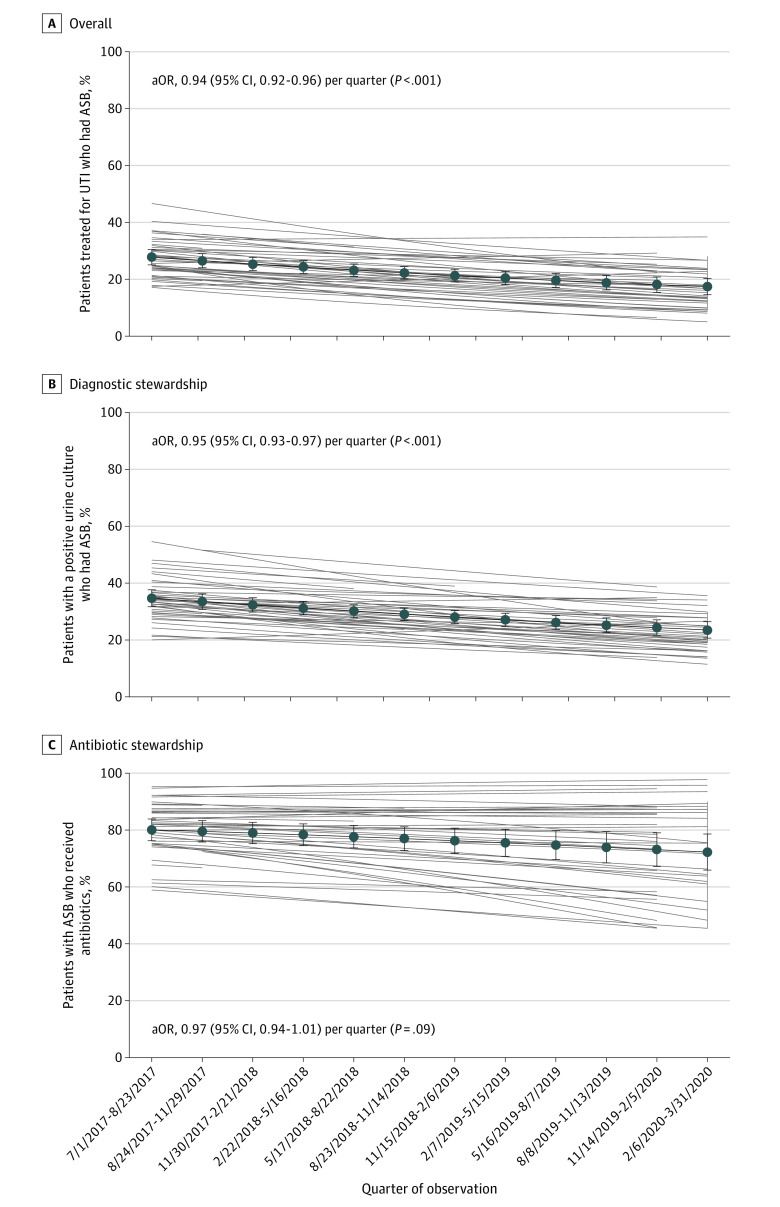
Diagnostic and Antibiotic Stewardship Outcomes Over Time Among 14 572 Patients Across 46 Hospitals Error bars represent 95% CIs. aOR indicates adjusted odds ratio; ASB, asymptomatic bacteriuria; UTI, urinary tract infection.

To estimate the change over time in the primary outcomes, we used logistic regression (for dichotomous outcome) or negative binomial models (for duration), adjusted for within-hospital clustering by allowing for random intercepts and slopes, to obtain adjusted odds ratios (aORs) or adjusted incidence rate ratios assessing the change in each outcome by quarter. Because patient characteristics were used to define appropriateness, no patient-level adjustments were included. Random intercepts describe baseline differences in treatment between hospitals. Random slopes estimate how the rate of change in treatment over time varied across hospitals. Using these models, we graphically presented (Figure 2) the predicted probability of each outcome averaged over all hospitals, as well as the smoothed individual curves for each hospital. To estimate potential ASB treatment cases avoided, we used the first 4 quarters of data as a baseline period. Then, using a random intercept logistic regression model, we estimated the difference in every patient’s probability of having ASB in the baseline period vs the postbaseline period with the difference summed across all postbaseline patients to estimate ASB cases avoided. We estimated potentially saved antibiotic days by multiplying ASB cases avoided by median ASB treatment duration.

To assess whether hospital characteristics were associated with changes in ASB treatment over time, we added potential hospital-level explanatory variables (eg, bed size) to the logistic regression models and assessed for interaction effects with baseline rates or slopes. Change in the number of urine cultures reported in point-prevalence surveys over time was assessed using linear regression. Change in signs and symptoms over time in patients classified as having a UTI was assessed using logistic regression.

All statistical tests were performed at an α level of .05. Two-tailed estimates of effect and 95% CIs are reported for all regression coefficients. Statistical analyses were performed with SAS, version 9.4 (SAS Institute), and StataSE, version 17 (StataCorp). Because disparities due to patient demographics may exist, we report data on sex, race, and ethnicity obtained from medical records and categorized as noted in the eAppendix in Supplement 1.

## Results

### Hospitals and Patient Population

Although 50 hospitals contributed data to HMS, 4 were excluded for having fewer than 10 patients, leaving 46 hospitals. After exclusions (eFigure 2 in Supplement 1), there were 14 572 included patients, of whom 28.4% (n = 4134) had ASB (range across hospitals, 16.4%-48.7%). Patient characteristics are summarized in Table 1. Missing data were uncommon and existed for 4.1% of patients (580 missing payer information and 54 missing race).

**Table 1.** Characteristics of Included Patients With UTI or ASB by Receipt of Antibiotic Therapy (N = 14 572 Patients Across 46 Hospitals)^a^

Patient characteristic	No. (%)		
	UTI treated with antibiotics (n = 10 438)	ASB treated with antibiotics (n = 3175)	ASB not treated with antibiotics (n = 959)
Sex			
Female	7184 (68.8)	2394 (75.4)	697 (72.7)
Male	3250 (31.1)	780 (24.6)	262 (27.3)
Race			
American Indian	25 (0.2)	11 (0.3)	2 (0.2)
Asian	64 (0.6)	10 (0.3)	4 (0.4)
Black	2209 (21.2)	687 (21.6)	177 (18.5)
Native Hawaiian or Other Pacific Islander	17 (0.2)	4 (0.1)	4 (0.4)
White	7767 (74.4)	2357 (74.2)	749 (78.1)
Other	181 (1.7)	41 (1.3)	15 (1.6)
Unknown	175 (1.7)	65 (2.0)	8 (0.8)
Age, median (IQR), y	75.0 (63.1-84.5)	78.8 (68.0-86.9)	74.7 (63.2-84.3)
Age group, y			
≥65	7501 (71.9)	2558 (80.6)	686 (71.5)
≥80	3882 (37.2)	1480 (46.6)	337 (35.1)
Insurance status			
Private	1425 (13.7)	305 (9.6)	134 (14.0)
Medicare	7488 (71.7)	2550 (80.3)	687 (71.6)
Medicaid	998 (9.6)	195 (6.1)	86 (9.0)
Uninsured	105 (1.0)	9 (0.3)	10 (1.0)
Missing	422 (4.0)	116 (3.7)	42 (4.4)
Charlson Comorbidity Index, median (IQR)	3 (1-5)	3 (1-5)	3 (1-5)
Comorbidities			
Presence of indwelling urinary catheter at time of urine culture	1383 (13.3)	446 (14.1)	82 (8.6)
Kidney disease	4228 (40.5)	1325 (41.7)	398 (41.5)
Hemodialysis	156 (1.5)	47 (1.5)	17 (1.8)
Liver disease	626 (6.0)	172 (5.4)	68 (7.1)
Congestive heart failure	2356 (22.6)	829 (26.1)	311 (32.4)
COPD	1854 (17.8)	612 (19.3)	190 (19.8)
History of cancer	2107 (20.2)	631 (19.9)	217 (22.6)
Immune compromise^b^	363 (3.5)	99 (3.1)	35 (3.7)
Dementia	2090 (20.0)	832 (26.2)	127 (13.2)
Diabetes	4014 (38.5)	1208 (38.1)	387 (40.4)
Sepsis			
≥2 SIRS criteria	6138 (58.8)	643 (20.3)	237 (24.7)
Severe sepsis^c^	2412 (23.1)	0	0
Duration of hospitalization at time of urine culture collection, median (IQR), d	0 (0-0)	0 (0-1)	0 (0-1)
Ordered urine culture, No./total No. (%)^d^			
Emergency medicine physician	6632/8510 (77.9)	1686/2447 (68.9)	483/783 (61.7)
Other	1878/8510 (22.1)	761/2447 (31.1)	300/783 (38.3)

Abbreviations: ASB, asymptomatic bacteriuria; COPD, chronic obstructive pulmonary disease; SIRS, systemic inflammatory response syndrome; UTI, urinary tract infection.

^a^ Patients were considered to have ASB if, based on medical record review, they did not have signs or symptoms of a UTI as defined by national guidelines. Antibiotic therapy was defined as any antibiotic therapy for a UTI regardless of duration (patients with concomitant infections were excluded).

^b^ Defined as having chemotherapy administered within 30 days, positive for HIV with a CD4 count greater than 200 cells/mm^3^, receiving prednisone dose of 10 mg/d or more for at least 30 days (or equivalent corticosteroid dose), receiving biologic agents, or having congenital or acquired immunodeficiency.

^c^ Patients with severe sepsis (ie, ≥2 SIRS criteria plus evidence of end organ damage) who were treated for a UTI were considered, by definition, to have a UTI.

^d^ Only a subset of patients had these data collected.

**Figure 3.**
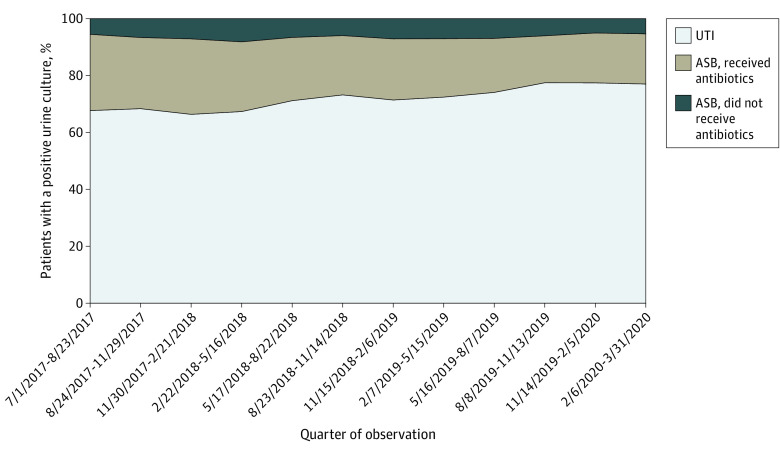
Change in Patient Categories Over Time Among 14 572 Patients Across 46 Hospitals ASB indicates asymptomatic bacteriuria; UTI, urinary tract infection.

Of all patients with ASB, 76.8% (3175 of 4134 patients) received antibiotic therapy (range across hospitals, 50.8%-100%). In patients with ASB who received antibiotic therapy, the median (IQR) duration was 6 (4-8) days. The most common empirical and discharge antibiotics are listed in eTable 1 in Supplement 1. Figure 3 shows the raw percentage of patients over time who had ASB treated with antibiotics, ASB not treated with antibiotics, or UTI.

Of the 46 included hospitals, 40 received the 2019 survey and 39 (98%) responded. An additional 3 hospitals answered a November 2018 survey when antibiotic stewardship, but not diagnostic stewardship, data were collected. All 42 responding hospitals had a UTI-directed antibiotic stewardship strategy during the study period, and 26 of 39 (66.7%) reported an ASB-directed diagnostic stewardship strategy. Additional self-reported diagnostic and antibiotic stewardship strategies are summarized in Table 2.

**Table 2.** Self-Reported Antibiotic and Diagnostic Stewardship Strategies (N = 42 Hospitals)^a^

Strategy	Hospitals responding yes, No. (%)
General stewardship infrastructure	
Has an antibiotic stewardship team	42 (100)
Stewardship resources increased since the Joint Commission standard^b^	12/39 (31)
Hospital policy requiring documentation of intended antibiotic duration	7 (17)
Antibiotic stewardship strategies	42 (100)
Antibiotic timeout at 48-72 h	13 (31)
All fluoroquinolones restricted	13 (31)
Institutional treatment guideline for UTI, updated within the past year	20 (48)
Indications of obtaining urine culture	18/20 (90)
Recommendations for not treating ASB	17/20 (85)
Antibiotic regimens that are concordant with national guidelines	20/20 (100)
Recommend against fluoroquinolone as first-line agent for cystitis	19/20 (95)
Clinicians educated on UTI and ASB	37 (88)
Audit and feedback for UTI	25 (60)
Audit and feedback for ASB	28 (67)
CPOE for UTI	28 (67)
CPOE for ASB	10 (24)
Diagnostic stewardship strategies (n = 39)^b^	26 (67)
No. of strategies (11 potential), median (IQR)	1 (0-2)
Removal/change in urine culture testing from preoperative order sets	3 (8)
Removal/change of urine culture testing from ED order sets	12 (31)
Removal/change of urine culture testing from admission order sets	7 (18)
Removal of urine culture testing from other order sets	6 (15)
Added reflex testing (urinalysis cutoff to urine cultures)	7 (18)
Removed reflex testing	7 (18)
Hiding urine culture results in some settings	1 (3)
Requiring physician order to run urine cultures in ED	6 (15)
2-Step urine culture initiative to reduce urine cultures in ED	3 (8)
Framing urine culture results in test results	3 (8)
Rejection of some urine cultures	3 (8)

Abbreviations: ASB, asymptomatic bacteriuria; CPOE, computerized provider order entry; ED, emergency department; UTI, urinary tract infection.

^a^ Responses are self-reported from a survey administered electronically using Qualtrics XM and emailed to all Michigan Hospital Medicine Safety Consortium hospitals in November 2019 (completed by December 2019). Hospitals were asked, “What strategies to reduce inappropriate use of urine cultures (e.g., diagnostic stewardship) has your hospital started?” (more details in the eAppendix in Supplement 1). The data abstractor (typically a nurse in quality) at each hospital was responsible for working with local individuals (eg, antibiotic stewardship leaders) to ensure survey accuracy and completion. Of the 46 study hospitals, only 40 received the 2019 survey; of those, 98% (n = 39) responded. An additional 3 hospitals that did not receive the 2019 survey answered a similar November 2018 survey when antibiotic stewardship, but not diagnostic stewardship data, were collected.

^b^ Not included in 2018 survey. Only 39 hospitals responded.

### Primary Outcomes

After adjusting for clustering, overall ASB-related antibiotic use decreased over the study period, illustrated by the percentage of patients treated for UTI who had ASB declining from 29.1% (95% CI, 26.2%-32.2%) to 17.1% (95% CI, 14.3%-20.2%) (aOR, 0.94 per quarter; 95% CI, 0.92-0.96; *P* < .001; Figure 2A). Compared with baseline, it was estimated that 590 ASB cases were avoided or there were 3540 fewer unnecessary days of antibiotic therapy. Regarding diagnostic stewardship, the percentage of patients with a positive urine culture who had ASB declined from 34.1% (95% CI, 31.0%-37.3%) to 22.5% (95% CI, 19.7%-25.6%) (aOR, 0.95 per quarter; 95% CI, 0.93-0.97; *P* < .001; Figure 2B). Regarding antibiotic stewardship, the percentage of patients with ASB who were treated with antibiotics remained stable over the study period, from 82.0% (95% CI, 77.7%-85.6%) to 76.3% (95% CI, 68.5%-82.6%) (aOR, 0.97 per quarter; 95% CI, 0.94-1.01; *P* = .09; Figure 2C). The mean duration of antibiotic therapy for ASB decreased only slightly from 6.38 days (95% CI, 6.00-6.78 days) to 5.93 days (95% CI, 5.54-6.35 days) (adjusted incidence rate ratio, 0.99 per quarter; 95% CI, 0.99-1.00; *P* = .045).

### Additional Analyses

No hospital characteristics were associated with baseline or change over time in the diagnostic stewardship metric (eTable 2A in Supplement 1). Although the average hospital did not see a reduction over time in the antibiotic stewardship metric, this was not true for all hospitals: independent hospitals and those belonging to state health care systems saw a larger slope decrease, which, for independent hospitals, could be because they had higher baseline ASB treatment (eTable 2B in Supplement 1).

Thirty-six hospitals reported data for urine culture prevalence surveys. The number of eligible urine cultures decreased over time (June 14-27, 2018: mean [SD], 15.8 [14.8]; April 4-17, 2019: mean [SD], 13.6 [13.0]; and April 28 to May 11, 2022: mean [SD], 11.6 [16.2]; *P* = .02; eFigure 3 in Supplement 1). This decrease in urine cultures occurred while bed size was stable (eFigure 4 in Supplement 1).

For patients classified as having a UTI (n = 10 438), there was no change over time in the frequency of objective signs (aOR per quarter of having any objective sign, 0.99; 95% CI, 0.97-1.00; *P* = .11; 84.0% in the first quarter to 82.0% in the last quarter). There was a small increase in subjective symptoms over time (aOR per quarter of having any subjective symptom, 1.03; 95% CI, 1.00-1.05; *P* = .04; 91.8% in the first quarter to 93.7% in the last quarter) (eAppendix in Supplement 1).

## Discussion

In this quality improvement study of 14 572 patients with a positive urine culture hospitalized at 46 hospitals, we found a decrease over time in overall ASB-related antibiotic use with an estimated 590 treated ASB cases and 3540 unnecessary days of antibiotic therapy avoided. Diagnostic, rather than antibiotic, stewardship appeared responsible for the improvement.

We found that the percentage of patients with a positive urine culture who had ASB decreased over time, suggesting unnecessary urine cultures decreased over time. In contrast, the percentage of patients with ASB who received antibiotics did not decrease over time, and antibiotic duration for ASB showed a clinically minor (approximately 0.04 days per quarter) decrease. Together, this suggests that strategies to reduce unnecessary urine cultures (ie, diagnostic stewardship) resulted in the majority of the reduction in overall ASB-related antibiotic use. The idea of diagnostic stewardship was first codified in 2017 by Morgan et al in a *JAMA* Viewpoint that described approaches to improving the use of diagnostic tests, such as urine cultures.^13^ The believed mechanism behind diagnostic stewardship is that a positive test, regardless of the positive predictive value of that result, serves as a powerful nudge for action—in this case antibiotic treatment.^15^ Thus, diagnostic stewardship works in part by reducing tests whose results are likely to be false positives. Evidence of the power of diagnostic over antibiotic stewardship for ASB is exemplified in 2 contrasting studies that attempted to reduce antibiotic use associated with ASB.^19,20^ In the first, antibiotic stewards conducted audit and feedback of newly reported positive urine cultures; even though stewards were able to intervene prior to initiation of antibiotics, this antibiotic stewardship strategy was not able to reduce antibiotic initiation for ASB.^20^ In contrast, hiding positive urine culture results from clinicians reduced antibiotic initiation for ASB from 48% to 12%.^19^ Multiple additional quasi-experimental studies have suggested diagnostic stewardship can be particularly influential for avoiding unnecessary antibiotic use for ASB.^15^ The present study adds to that literature by finding that, across diverse hospitals, diagnostic stewardship is most associated with improved ASB treatment. Even though fewer hospitals reported using a diagnostic stewardship strategy for ASB, it appears to be the more effective strategy.

### Strengths and Limitations

This study must be interpreted in the context of limitations. First, due to heterogeneity in type, strength, and timing of diagnostic stewardship strategies reported by hospitals, we were unable to assess which diagnostic stewardship strategy was most effective. Second, we were unable to collect complete data on the number of urine cultures over time. The findings from the 3 prevalence surveys that the number of patients with positive urine cultures decreased despite stable hospital bed size supports the inference that urine cultures decreased over time. Third, we were unable to collect data on hospitalized patients in whom urine cultures were avoided. It is possible that these patients still received antibiotic therapy. Fourth, we relied on data collection from medical records. It is possible that documentation of symptoms increased over time (improved documentation vs gaming), which could affect the classification of UTI vs ASB over time. However, in a sensitivity analysis, we found that most objective signs of a UTI (leukocytosis, hypotension, SIRS criteria) remained stable over time, suggesting documentation did not substantially influence the changes seen. Study strengths include, to our knowledge, the largest sample size, including medical record review, for an ASB study; a large, diverse group of hospitals; and the ability to distinguish the outcomes of diagnostic vs antibiotic stewardship over 3 years.

This study has implications. Antibiotic stewardship is a Joint Commission and Centers for Medicare & Medicaid Services requirement for accreditation/participation. We found that diagnostic stewardship may improve ASB-related antibiotic use more than antibiotic stewardship. Although we could not assess which specific diagnostic stewardship strategy was most effective, best practice may be to identify where (eg, emergency department vs preoperative) and how (eg, automatic through order sets, nursing-driven protocol) inappropriate urine cultures are being ordered and design a diagnostic stewardship strategy based on need. Thus, national policy and antibiotic stewardship programs should consider requiring and incorporating diagnostic stewardship into ASB improvement efforts.

## Conclusions

In this quality improvement study, we found that antibiotic treatment for ASB appeared to decrease over time, associated with a reduction in unnecessary urine cultures. Hospitals and national policy should prioritize reducing unnecessary urine cultures (ie, diagnostic stewardship) to reduce ASB-related antibiotic use.

## Notes

Supplement 1. **eFigure 1.** Timeline of Michigan Hospital Medicine Safety Consortium Events
eAppendix
**eFigure 2.** Study Flow Diagram **eTable 1.** Antibiotic Treatment in Patients With UTI or ASB Who Were Treated With Antibiotics **eTable 2A.** Association of Hospital Characteristics With Baseline Rate and Change in Diagnostic Stewardship Metric (Percentage of Hospitalized Patients With a Positive Urine Culture Who Had ASB) **eTable 2B.** Association of Hospital Characteristics With Baseline Rate and Improvement in Antibiotic Stewardship Metric (Percentage of ASB Patients Who Were Treated with Antibiotics) **eFigure 3.** Urine Culture Two Week Prevalence Surveys, n=39 Hospitals **eFigure 4.** Distribution of Hospital Bed Size Over Time, n=39 Hospitals

Supplement 2. Data Sharing Statement
