# Supplementary material for: A Statewide Quality Initiative to Reduce Unnecessary Antibiotic Treatment of Asymptomatic Bacteriuria
Source: JAMA Intern Med. 2023 Jul 10;183(9):933–41. doi: 10.1001/jamainternmed.2023.2749 (PMC10334295; doi:10.1001/jamainternmed.2023.2749)
Supplement: Supplement 1. — eFigure 1. Timeline of Michigan Hospital Medicine Safety Consortium Events eAppendix eFigure 2. Study Flow Diagram eTable 1. Antibiotic Treatment in Patients With UTI or ASB Who Were Treated With Antibiotics eTable 2A. Association of Hospital Characteristics With Baseline Rate and Change in Diagnostic Stewardship Metric (Percentage of Hospitalized Patients With a Positive Urine Culture Who Had ASB) eTable 2B. Association of Hospital Characteristics With Baseline Rate and Improvement in Antibiotic Stewardship Metric (Percentage of ASB Patients Who Were Treated with Antibiotics) eFigure 3. Urine Culture Two Week Prevalence Surveys, n=39 Hospitals eFigure 4. Distribution of Hospital Bed Size Over Time, n=39 Hospitals [file jamainternmed-e232749-s001.pdf]

Supplemental Online Content

Vaughn VM, Gupta A, Petty LA, et al. A statewide quality initiative to reduce unnecessary antibiotic treatment of asymptomatic bacteriuria. *JAMA Intern Med*. Published online July 10, 2023. doi:10.1001/jamainternmed.2023.2749

eFigure 1. Timeline of Michigan Hospital Medicine Safety Consortium Events
eAppendix
eFigure 2. Study Flow Diagram
eTable 1. Antibiotic Treatment in Patients With UTI or ASB Who Were Treated With Antibiotics
eTable 2A. Association of Hospital Characteristics With Baseline Rate and Change in Diagnostic Stewardship Metric (Percentage of Hospitalized Patients With a Positive Urine Culture Who Had ASB)
eTable 2B. Association of Hospital Characteristics With Baseline Rate and Improvement in Antibiotic Stewardship Metric (Percentage of ASB Patients Who Were Treated with Antibiotics)
eFigure 3. Urine Culture Two Week Prevalence Surveys, n=39 Hospitals
eFigure 4. Distribution of Hospital Bed Size Over Time, n=39 Hospitals

This supplemental material has been provided by the authors to give readers additional information about their work.

© 2023 Vaughn VA et al. *JAMA Intern Med.*

eFigure 1. Timeline of Michigan Hospital Medicine Safety Consortium Events

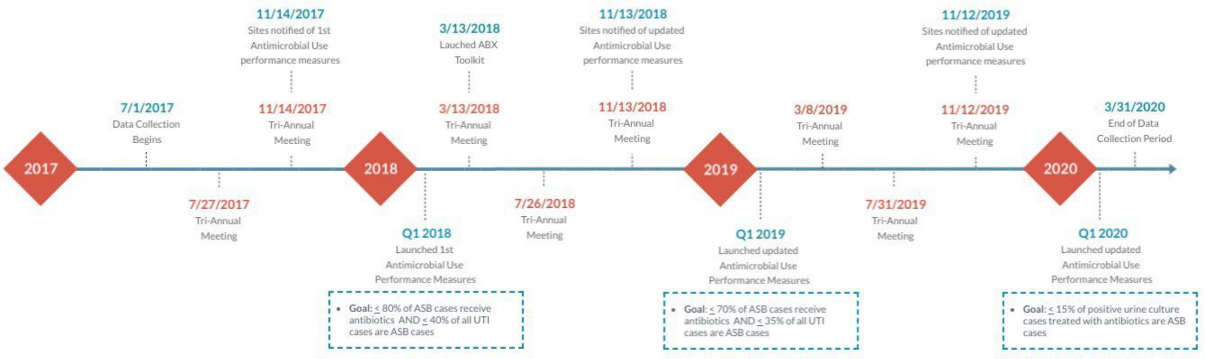

© 2023 Vaughn VA et al. *JAMA Intern Med.*

eAppendix.

HMS Pillar One:
Data Sharing and Benchmarking

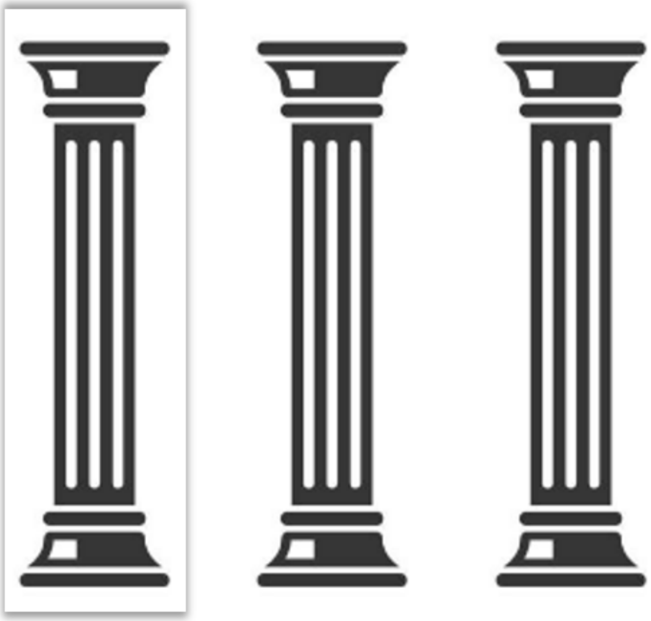

© 2023 Vaughn VA et al. *JAMA Intern Med.*

Use of Antibiotics in Patients with ASB Rates: Raw vs. Adjusted

HMS 2022 Performance Index Measure #7: Reduce Use of Antibiotics in Patients with ASB

SAMPLE

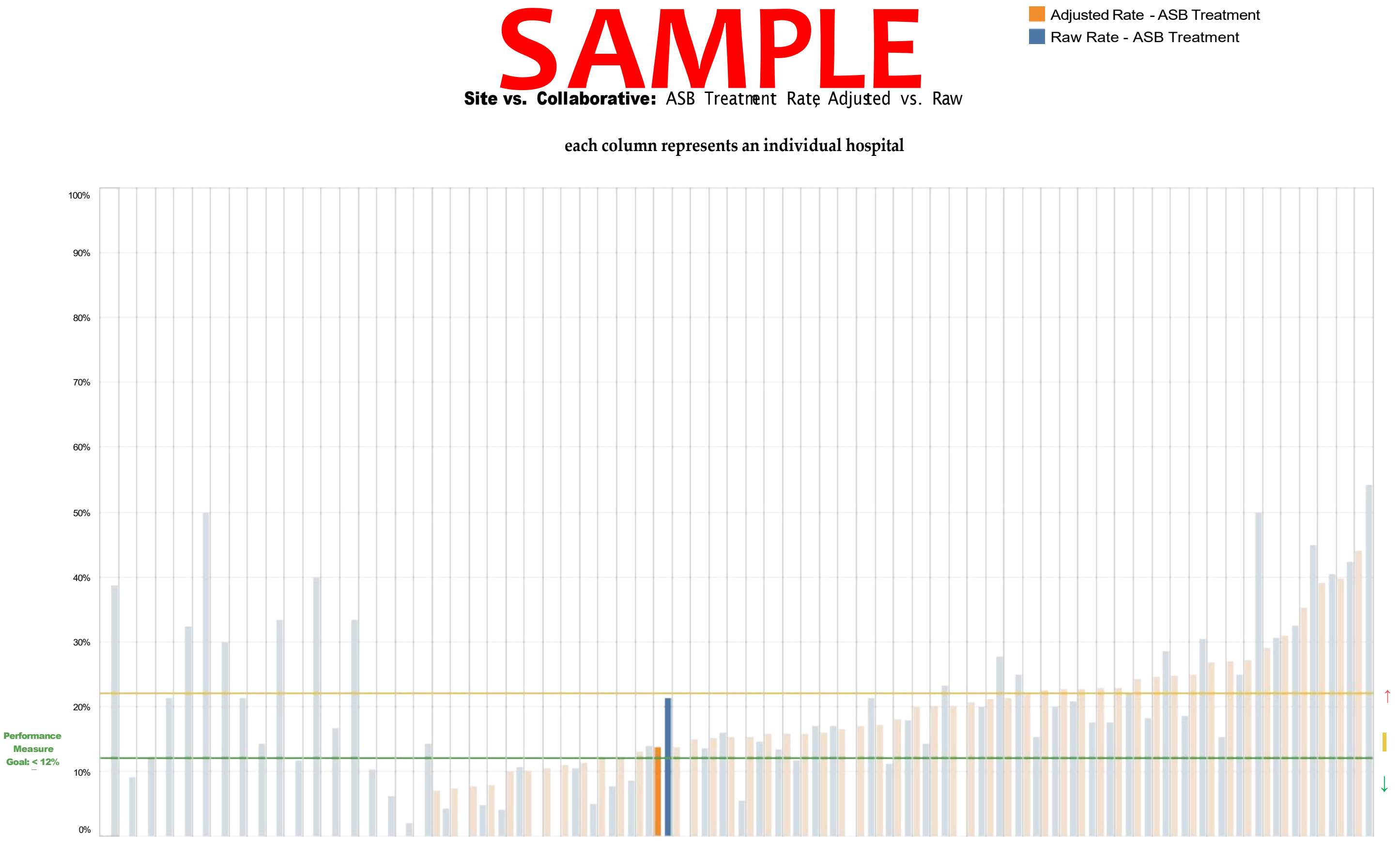

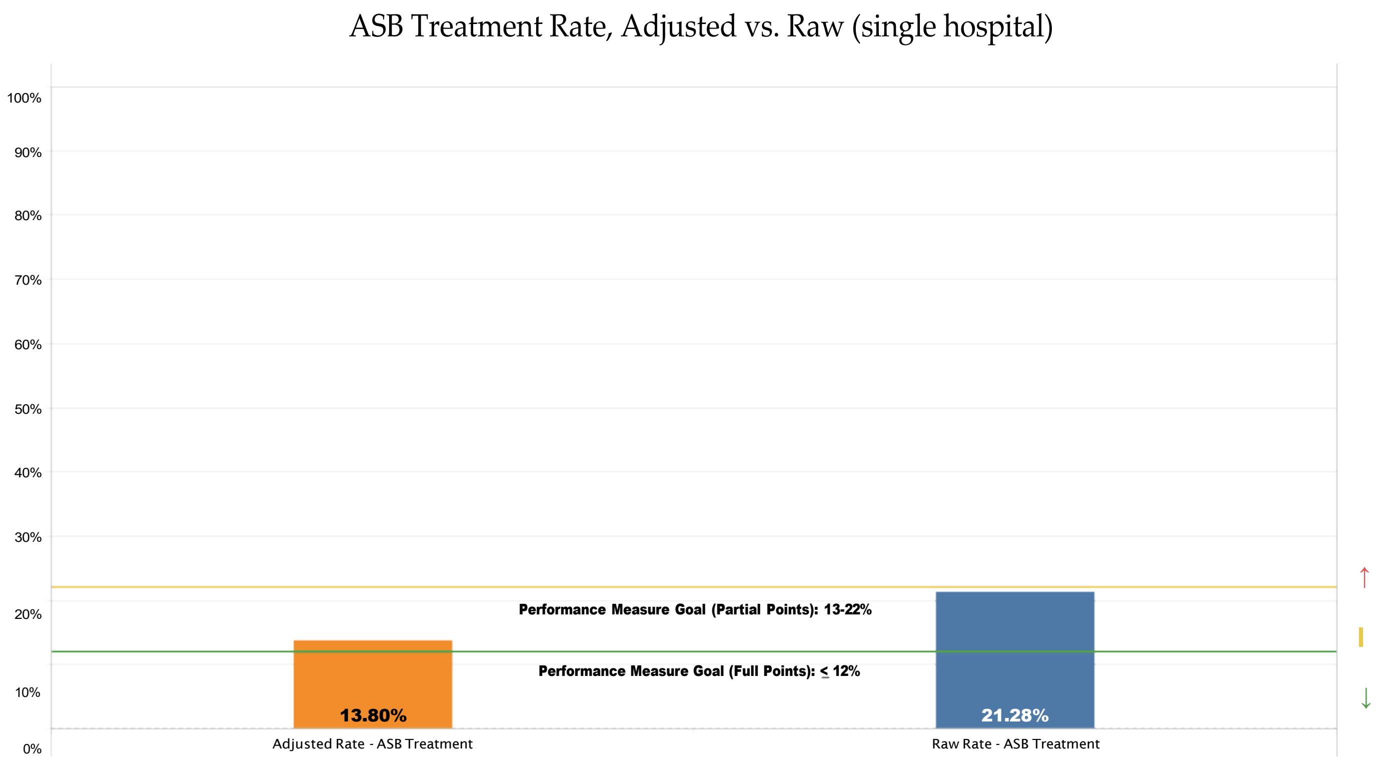

© 2023 Vaughn VA et al. *JAMA Intern Med.*

HMS - Antimicrobial Use Report - Q2 2022
July 2022

SAMPLE

Sample Site Report

Discharge Dates: 02/17/2022 - 05/11/2022

Date of data pull: 6/24/2022

Completed Cases entered during reporting period (n)

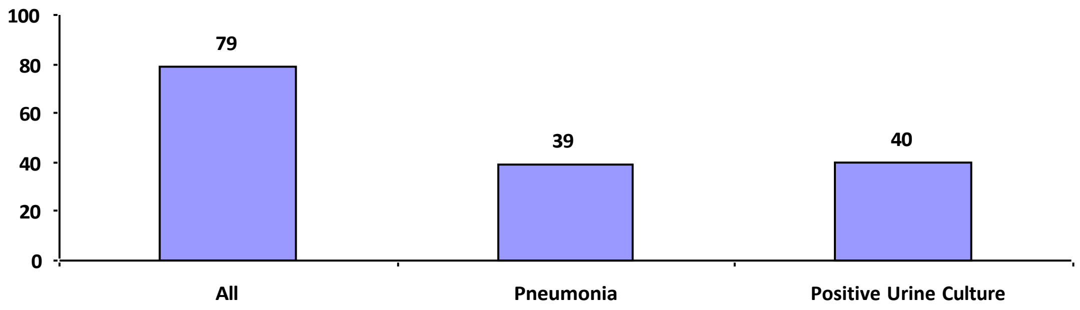

Number of completed cases with clean data: 79

ID of cases removed from report:[1] N/A

Follow up information obtained during phone call (Of those eligible for follow up phone call):[2] 49/65 (75.4%)

© 2023 Vaughn VA et al. *JAMA Intern Med.*

HMS - Antimicrobial Use Report - Q2 2022
July 2022

Positive Urine Culture Cohort

Positive Urine Culture Cohort Category Distribution[1,2]

Total: 40

Inpatient LOS - Median:3

% cases with NHSN CAUTI: 1/40 (2.5%)

% ASB cases: 10/40 (25%)

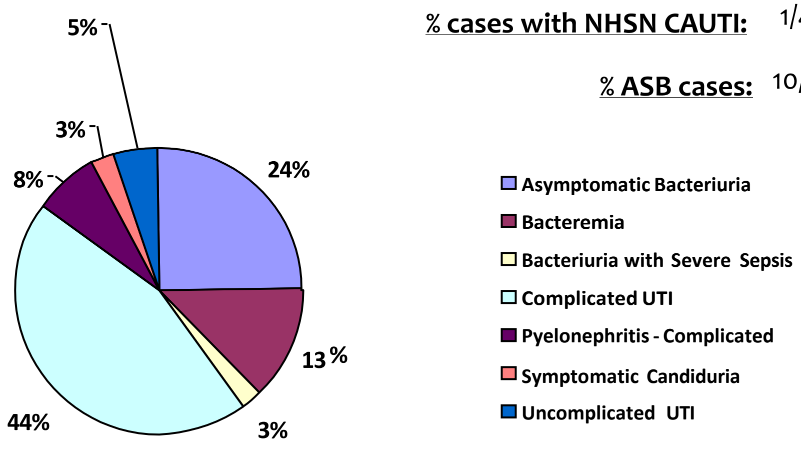

Asymptomatic Bacteriuria HMS ID: in an actual report, case numbers would be provided

Median Duration of antibiotics for cases that received treatment for a positive urine culture[3]

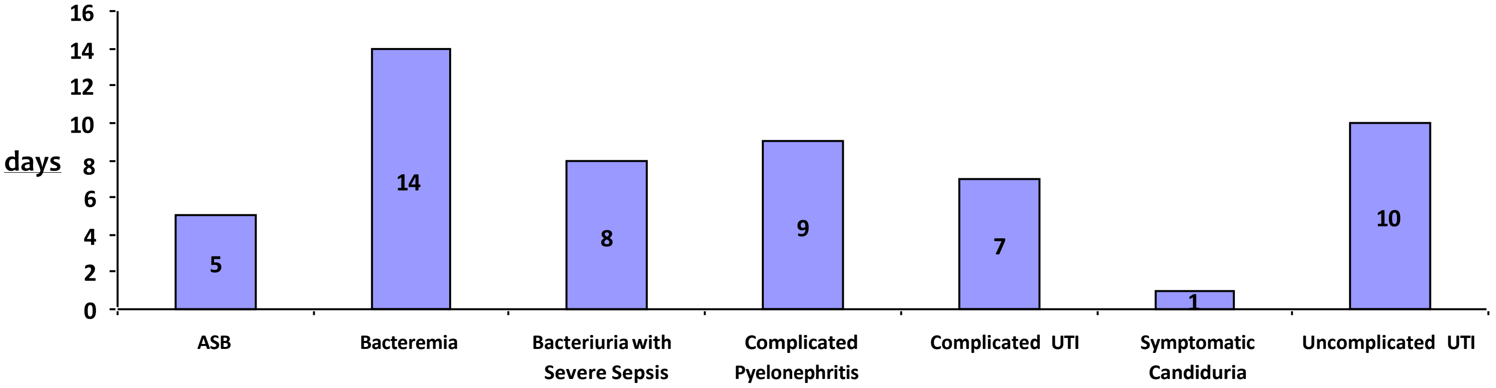

Footnotes:

1) See page 14 for definitions
2) Cases where there is a positive blood culture but the pathogen in the positive blood culture is not one of the pathogens in the positive urine culture are not included in category determination and metrics.
3) ASB Includes Yeast. Symptomatic Candiduria only includes antibacterial agents.

© 2023 Vaughn VA et al. *JAMA Intern Med.*

HMS - Antimicrobial Use Report - Q2 2022 July 2022

Positive Urine Culture Cohort[1]

Percent of cases that received Antibiotics

ASB
(Antibiotics Day 1 or Later)

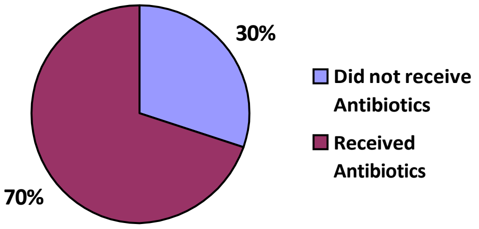

ASB
(Antibiotics Day 2 or Later)

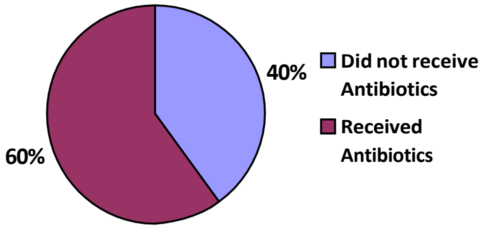

Treated ASB (Day 2 or Later) Out of All Positive Urine Culture Cohort

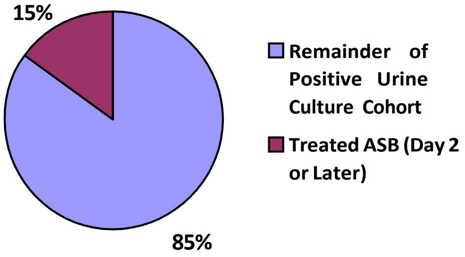

Performance Metric

HMS ID - Treated ASB (Day 2 or Later) Out of All Positive Urine Culture Cohort:
in an actual report, case numbers would be provided for hospitals to review

% Received Excess Duration of Therapy

Number of cases	6	5	1
Median abx duration	6	9	13

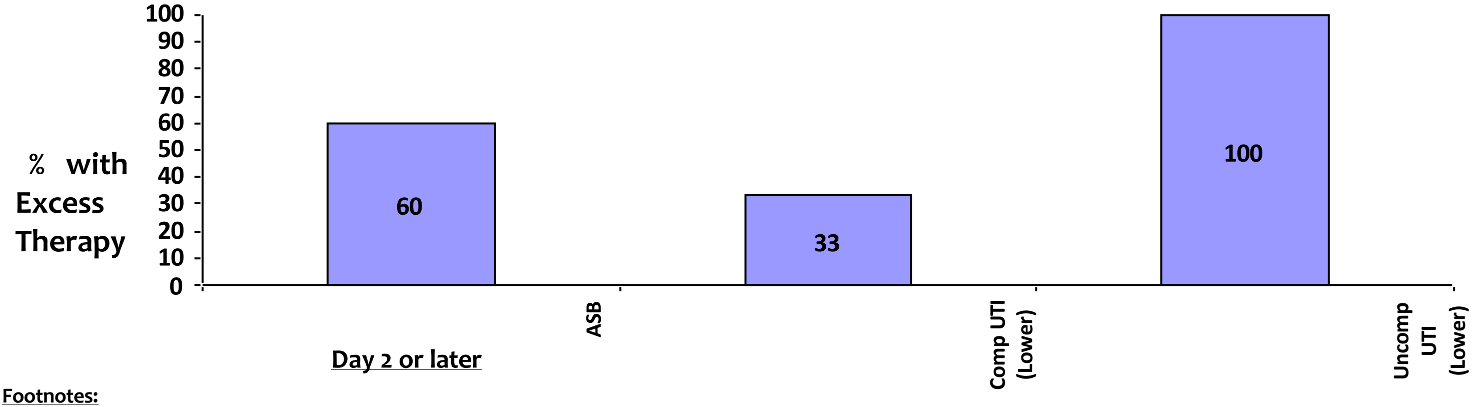

Footnotes:
1) Cases where there is a positive blood culture but the pathogen in the positive blood culture is not one of the pathogens in the positive urine culture are not included in category determination and metrics.

© 2023 Vaughn VA et al. *JAMA Intern Med.*

HMS - Antimicrobial Use Report - Q2 2022
July 2022

Positive Urine Culture Cohort

ASB CASES ONLY[1]

Who ordered the urine culture

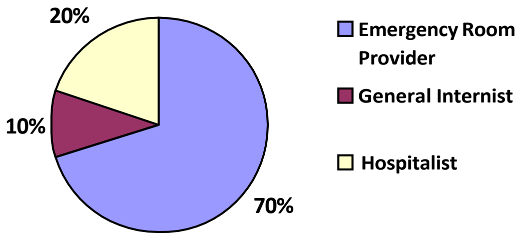

Who ordered the antibiotics

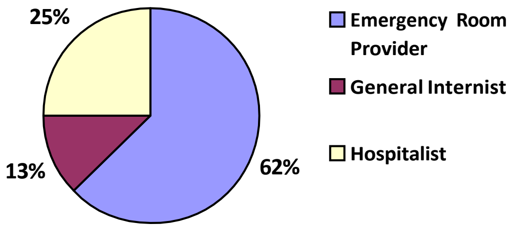

Received Fluoroquinolone[2,3]

Received Non-Preferred Fluoroquinolone

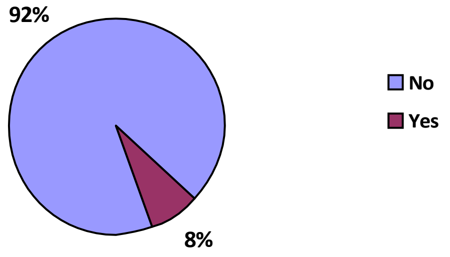

Performance Measure

Breakdown of cases receiving Non-Preferred Fluoroquinolone

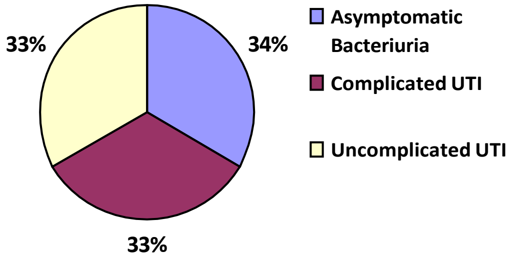

HMS ID of cases with Non-Preferred FQ use: in an actual report, case numbers would be provided for hospitals to review

Footnotes:

1) "Who ordered the urine culture" is at the patient level. "Who ordered antibiotics" is at the antibiotic level.
2) Fluoroquinolone use is reflective of inpatient or discharge treatment (ciprofloxacin or levofloxacin)
3) Non preferred Fluoroquinolone use is either due to treatment of Asymptomatic Bacteriuria (ASB) or treatment of UTI when there is a safer alternative. For this metric, fluoroquinolone use in bacteremia and pyelonephritis is considered acceptable.

© 2023 Vaughn VA et al. *JAMA Intern Med.*

HMS - Antimicrobial Use Report - Q2 2022 July 2022

MICHIGAN HOSPITAL MEDICINE SAFETY CONSORTIUM

Positive Urine Culture Cohort

Document Indication and Duration of Antibiotics for Positive Urine Culture Cases

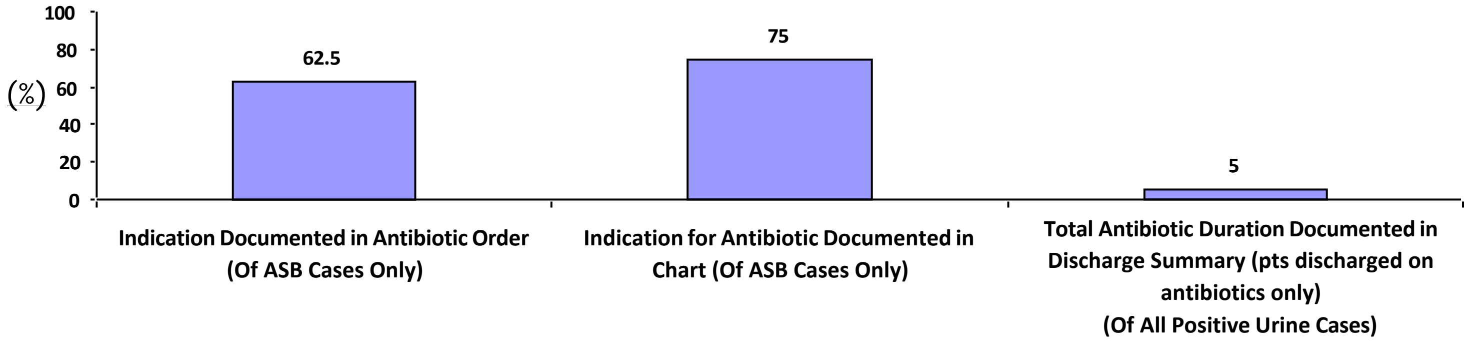

Top 5 Antibiotics for all cases treated

Inpatient ABX x% (n)	Discharge ABX x% (n)
1. Ceftriaxone 82.9% (29)	1. Cephalexin 30.4% (7)
2. Cefepime 22.9% (8)	2. Ciprofloxacin/Levofloxacin 26.1% (6)
3. Cephalexin 14.3% (5)	3. Cefpodoxime 13% (3)
4. Vancomycin 14.3% (5)	4. Bactrim 8.7% (2)
5. Ciprofloxacin/Levofloxacin 11.4% (4)	5. Cefuroxime 8.7% (2)

Top 5 Antibiotics for ASB cases treated

Inpatient ABX x% (n)	Discharge ABX x% (n)
1. Ceftriaxone 87.5% (7)	1. Cefuroxime 33.3% (1)
2. Vancomycin 12.5% (1)	2. Cephalexin 33.3% (1)
3.	3. Ciprofloxacin/Levofloxacin 33.3% (1)
4.	4.
5.	5.

© 2023 Vaughn VA et al. *JAMA Intern Med.*

HMS - Antimicrobial Use Report - Q2 2022 July 2022

HMS MICHIGAN HOSPITAL MEDICINE SAFETY CONSORTIUM

Positive Urine Culture Cohort

Pathogens - All Categories (%) -Excludes Pyelonephritis/Bacteremia[1]

N: 32

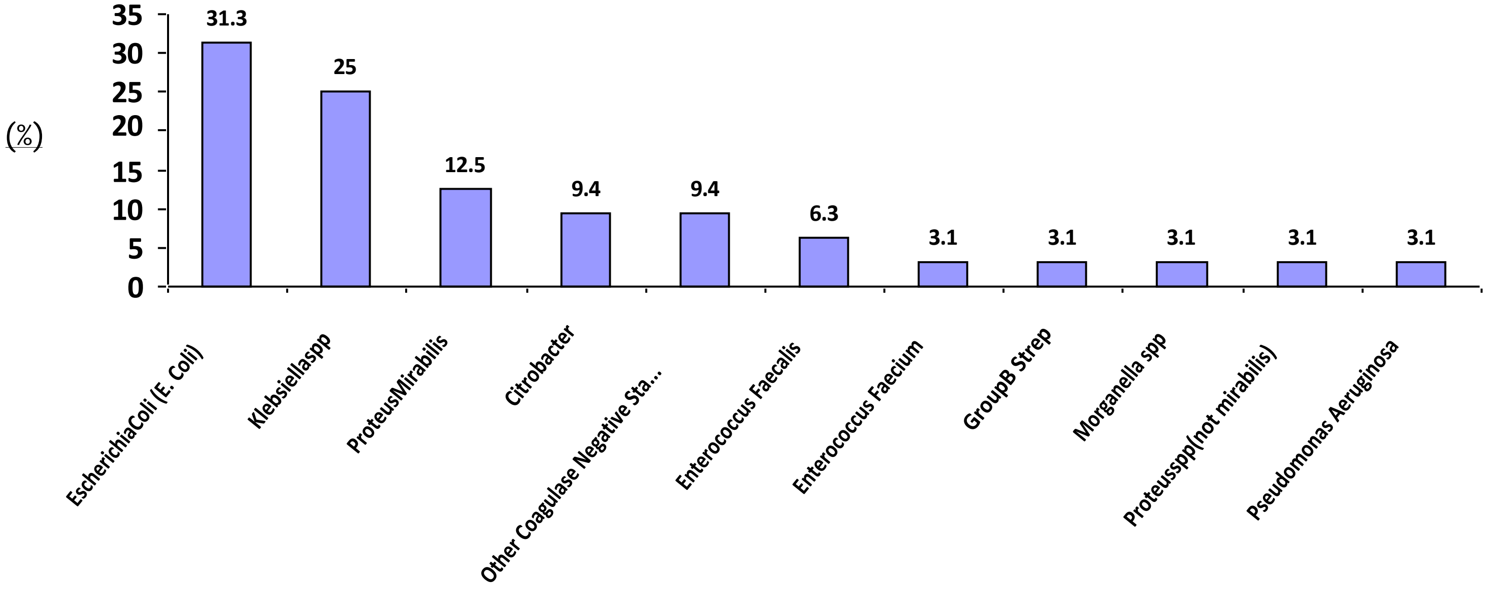

Pathogens - Complicated UTI (%) Excludes Pyelonephritis/Bacteremia[1]

N: 18

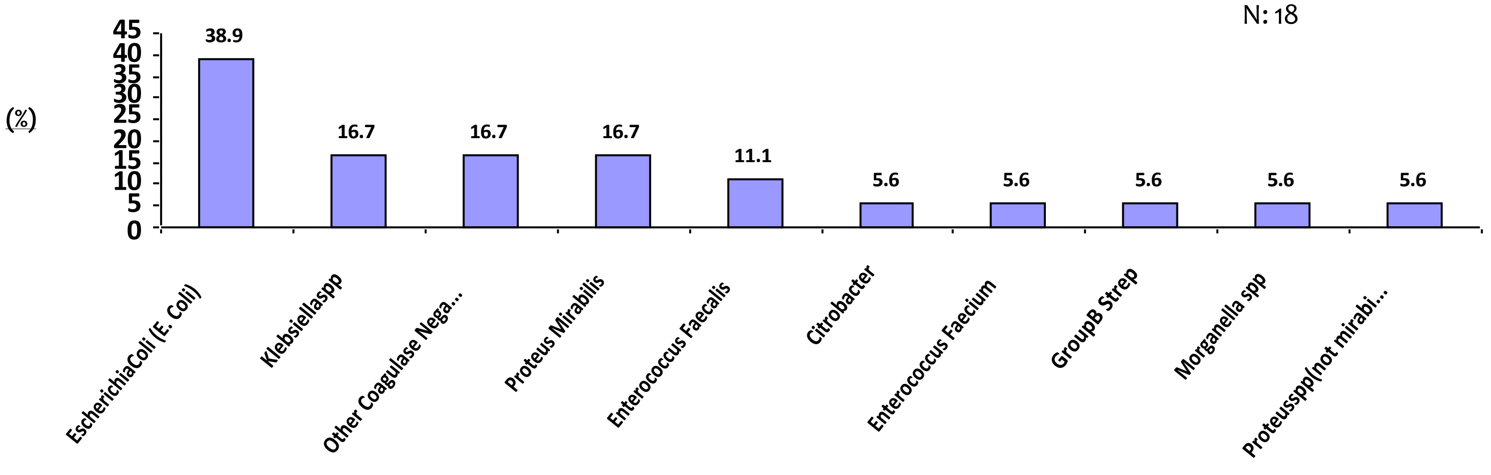

Footnotes:

1) Cases can have more than 1 pathogen

© 2023 Vaughn VA et al. *JAMA Intern Med.*

HMS - Antimicrobial Use Report - Q2 2022 July 2022

Positive Urine Culture Cohort

Adverse Events for Positive Urine Cases

30-Day Readmission Rate: 7/40 (17.5%) 30-Day Mortality Rate: 2/40 (5%)

Emergency visit in 30 days (without admission): 11/40 (27.5%)

Outpatient visit in 30 days: 6/40 (15%)

Adverse events due to antibiotics for Positive Urine Cases[1]

Clostridiodes Difficile:[1] 1/40 (2.5%)

HMS ID - Clostridiodes Difficile Documented: in an actual report, case numbers would be provided and patients who received fluoroquinolone would be marked with *

Allergy/Adverse Event due to Antibiotics (Physician/Patient Reported) e.g., nausea, leukopenia: 1/40 (2.5%)

HMS ID - Adverse Event: in an actual report, case numbers would be provided for hospital review

Footnotes:

1) Includes index hospitalization + 30 days

© 2023 Vaughn VA et al. *JAMA Intern Med.*

HMS Pillar Two:
Sharing Best Practices

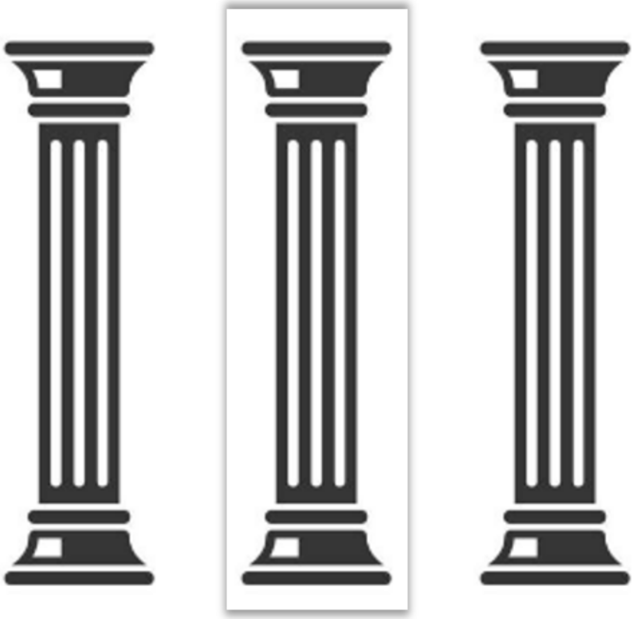

© 2023 Vaughn VA et al. *JAMA Intern Med.*

Example Information From HMS Antimicrobial Initiative Toolkit

**2. Develop and Share Institutional Guidelines for UTI/ASB**	● Develop institutional guidelines, locally-adapted from national and HMS guidelines, for urinary tract infection (UTI)/asymptomatic bacteriuria (ASB). If institution specific guidelines already exist, they should comply with the following:<br>▪ **UTI/ASB**<br>*Institutional guidelines should:*<br>▪ Recommend against sending urine cultures in the absence of urinary symptoms<br>▪ Recommend against treating a positive urine culture in the absence of urinary symptoms<br>▪ De-emphasize fluoroquinolones<br>▪ Provide recommendations for transition to oral therapy<br>● Share the UTI/ASB guidelines with members of the work group and frontline providers to get feedback and to ensure buy-in.<br>● Publish guidelines in multiple formats, including booklet, hospital intranet, or an application for smartphones.	**Resources & Tools:**<br>*Examples of Guidelines that could be locally-adapted to your institution:*<br>● National Guidelines:<br>▪ IDSA Guidelines for the Diagnosis and Treatment of Asymptomatic Bacteriuria in Adults. CID 2019.<br>▪ IDSA and European Society for Microbiology and Infectious Disease Guidelines for Treatment of Acute Uncomplicated Cystitis and Pyelonephritis in Women. CID 2010.<br>▪ IDSA Guidelines for Diagnosis, Prevention, and Treatment of Catheter-Associated Urinary Tract Infection (CA-UTI) in Adults. CID 2010.<br>● HMS Guideline:<br>▪ UTI<br>● Institutional Guideline Examples:<br>▪ UTI Guideline Example #1<br>▪ UTI Guideline Example #2<br>● Pocket Cards:<br>▪ CAP<br>▪ UTI

**3. Integrate and Operationalize Institutional Guidelines for UTI and CAP**	● Educate providers, including hospitalists, internal medicine, family medicine, emergency medicine physicians, residents, advanced practice professionals (APPs), and nursing staff about antibiotic resistance and appropriate antimicrobial prescribing.<br>● Educate patients and families about antibiotic resistance and appropriate antimicrobial prescribing.<br>● During educational sessions, highlight HMS data, showing opportunities for improvement.<br>● Communicate and promote institution-specific guidelines with frontline providers, including physicians, APPs, nursing, and pharmacy to ensure use of recommendations (morning report, grand rounds, medical staff meetings, division meetings).<br>● Integrate recommendations into key processes within the healthcare system such as into order sets, individual orders, discharge planning/processes, required yearly education for staff, etc.<br>● Build systems that can help modify provider behavior. Examples include (but are not limited to): clinical decision support tools and pharmacist review of antibiotic prescribing.<br>● After 3 months of guideline use, obtain provider feedback from multiple groups (including hospitalists, internal medicine, emergency department, etc.), and modify accordingly.<br>[illegible]	**Resources & Tools:**<br>● Review HMS institution specific data to identify areas for local improvement<br>● UTI Order Set Example (Michigan Medicine)<br>● Patient Education Handout Example: What You Need to Know When You are Prescribed an Antibiotic<br>● Presentations (For HMS Members):<br>▪ Henry Ford Antimicrobial Stewardship Team at the March 21, 2018 HMS Collaborative Wide Meeting: *Implementation of a Short Course Antibiotic Initiative*<br>▪ Dr. Sara Cosgrove (Director of the Antimicrobial Stewardship Program, Johns Hopkins Hospital) at the July 12, 2018 HMS Collaborative Wide Meeting: *Optimizing Antimicrobial Use in the Inpatient Setting*<br>▪ Dmitriy Martirosov (Infectious Disease Pharmacist, Beaumont Health System) at the March 8, 2019 HMS Collaborative Wide Meeting: *Curtailing Diagnosis and Treatment of Asymptomatic Bacteriuria (ASB)*<br>▪ Dr. Larissa May (Director of Emergency Department Antibiotic Stewardship, University of California-Davis) at the November 12, 2019 HMS Collaborative Wide Meeting: *Doing What's Best for Our Patients: Antibiotic Stewardship in the ED Setting*<br>**References:**<br>● Ciarkowski CE et al. A Pathway for Community-Acquired Pneumonia with Rapid Conversion to Oral Therapy Improves Health Care Value. *Open Forum Infect Dis* 2020.

**5. Reduce Testing and Treatment of Asymptomatic Bacteriuria (ASB)**	● Educate providers, including hospitalists, internal medicine, family medicine, emergency medicine physicians, residents, advanced practice professionals (APPs), and nursing staff regarding the diagnosis of ASB vs UTI.	**Resources & Tools:**<br>● Review HMS site reports (hard copy distributed at collaborative wide meetings and live reports available daily via the HMS data entry system) for the following:<br>▪ Testing of Asymptomatic Bacteriuria

© 2023 Vaughn VA et al. *JAMA Intern Med.*

Improvement in ASB Testing: Diagnostic Stewardship

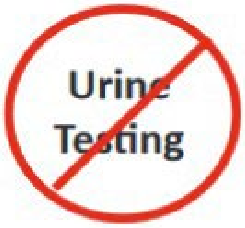

Do NOT Send Urinalysis or Urine Culture
if none of these symptoms are present or there is an alternative cause for the symptom

Signs & Symptoms without alternative cause
Fever >38° C or rigors
Urgency, frequency, dysuria
Suprapubic pain or tenderness
Costovertebral pain or tenderness
New onset mental status changes with leukocytosis (>10,000 cells/mm^3), hypo-tension (<90mmHg Systolic), or >/= 2 SIRS criteria[1]
Acute hematuria
Spasticity or autonomic dysreflexia in patients with spinal cord injury

HMS UTI Treatment Pocket Card

EMPIRIC THERAPY BASED ON CLASSIFICATION OF URINARY TRACT INFECTION

Empiric choices should take into account previous cultures, antibiotic allergies, local antibiotic susceptibilities, and severity of illness.
If urine culture is negative & patient was on antibiotics at the time of culture & patient has symptoms (1-7 on the reverse side), it may be appropriate to treat.

PATIENT CATEGORY	PREFERRED**	ALTERNATIVES	DURATION
*ASYMPTOMATIC BACTERIURIA** Defined as having NONE of the symptoms (1-7) listed on reverse side	Treatment indicated during pregnancy and prior to urologic procedures		
*UNCOMPLICATED LOWER UTI (CYSTITIS)****	Nitrofurantoin or TMP/SMX	Fosfomycin IV or Oral Beta-Lactam (e.g. Cephalexin or Cefpodoxime)	Nitrofurantoin x 5 days (avoid in CrCl < 30 mL/min)<br>Fosfomycin x 1 dose<br>TMP/SMX x 3 days<br>IV or Oral Beta-Lactam x 3-7 days
*COMPLICATED LOWER UTI (CYSTITIS)****<br>Male, urinary catheter present or within last 48 hours, anatomic abnormality or obstruction, significant co-morbidities	Nitrofurantoin, Fosfomycin, or TMP/SMX, Oral Beta-Lactam or IV Beta-Lactam, Severe PCN or Cephalosporin Allergy: Aztreonam		Nitrofurantoin x 7 days (avoid in CrCl < 30 mL/min)<br>Fosfomycin (q48h) x 3-5 doses<br>TMP/SMX x 7 days<br>Oral Beta-Lactam, IV Beta-Lactam, or Aztreonam x 7 days
UNCOMPLICATED PYELONEPHRITIS	TMP/SMX, Fluoroquinolones, or Beta-Lactams		IV Beta-Lactam Therapy followed by Oral Beta-Lactam or Oral TMP/SMX therapy : 7-14 days<br>IV Beta-Lactam Therapy x 7 days<br>TMP/SMX x 7-14 days<br>Fluoroquinolones x 5-7 days
COMPLICATED PYELONEPHRITIS, UTI WITH BACTEREMIA & SEPSIS	Defer to Individual Institutions		**Complicated Pyelonephritis** : 7-14 days<br>**UTI with Bacteremia** : 7-14 days<br>[Shorter courses of therapy (7 days) with a fluoroquinolone or IV beta-lactam can be considered in patients with uncomplicated bacteremia secondary to pyelonephritis or cystitis/lower UTI and have rapid clinical response to therapy.]

*refer to reverse side for conditions when symptom based screening may not be appropriate
**preferred therapies should reflect local antibiogram data for *E.coli* >80% susceptible
*** excludes patients with sepsis and bacteremia
Follow culture results and de-escalate therapy based on final results and sensitivities.
FOR EACH ANTIBIOTIC: DOCUMENT INDICATION AND PLANNED DURATION FOR ALL PATIENTS.
For more detail about these guidelines, please see the Guidelines for Treatment of UTIs published by HMS.

© 2023 Vaughn VA et al. *JAMA Intern Med.*

Educational Resources for Physicians and Nurses

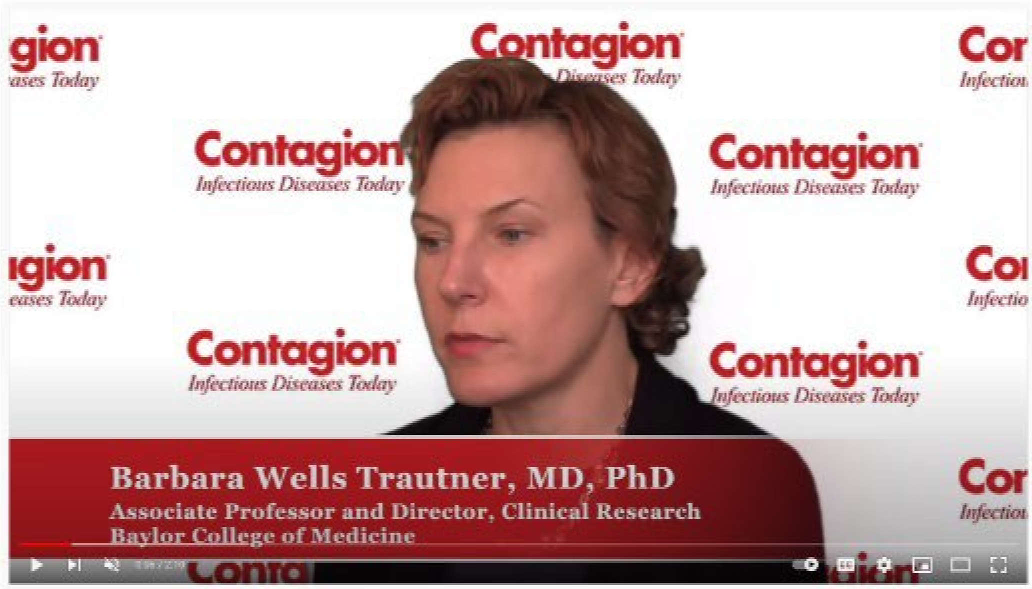

The Dangers of Overtreating Asymptomatic Bacteriuria

Treatment of Asymptomatic Bacteriuria: Impact of Educational Intervention and Pocket Card

All Patients	Baseline N= 162	Post educational intervention and pocket card N= 152	Change from baseline (%)
Patients with ASB	99 (61.1%)	92 (60.5%)	-0.6% (p=0.916)
Treatment rates of ASB	76/99 (76.8%)	49/92 (53.3%)	-23.5% (p=0.001)
Treatment rates for patients with ASB and guideline-based clinical manifestation from a condition other than UTI.	31/43 (72.1%)	30/51 (58.8%)	-13.3% (p=0.179)
Treatment rates for patients with ASB and no guideline-based clinical manifestations	45/56 (80.4%)	19/41 (46.3%)	-34.1% (p<0.001)
Antimicrobial days/patient with ASB	4.6 (455 days/99 patients)	3.3 (305 days/92 patients)	-28.3% (p<0.001)
**Patients with a Urinary Catheter**			
Patients with ASB and a urinary catheter	20/99 (20.2%)	29/92 (31.5%)	+11.3% (0.073)
Treatment rates for catheterized patients with ASB	15/20 (75.0%)	20/29 (69.0%)	-6.0% (p=0.646)
Treatment rates for catheterized patients with ASB and guideline-based clinical manifestation from a condition other than UTI	8/10 (80.0%)	14/18 (77.8%)	-2.2% (p=1.000)
Treatment rates for catheterized patients with ASB and no guideline-based clinical manifestation	7/10 (70.0%)	6/11 (54.5%)	-15.5 (p=0.659)
Antimicrobial days/catheterized patient with ASB	5.2 (104 days/20 patients)	4.7 (137 days/29 patients)	-9.6 % (p=0.460)

© 2023 Vaughn VA et al. *JAMA Intern Med.*

Assessment of Testing and Treatment of ASB Initiated in the Emergency Department

- Testing and treatment for ASB is often started in the Emergency Department
    - Partnership upcoming with the Michigan Emergency Department Improvement Collaborative
- Predictors of ED clinician treatment of ASB include:
    - Dementia
    - Spinal cord injury
    - Incontinence or presence of a urinary catheter
    - Altered mental status
    - Leukocytosis or abnormal urinalysis

- Once started on antibiotic treatment of ASB in the ED, 80% of patients remain on antibiotics for at least **3 days**
- Antibiotic treatment in these patients is associated with a longer length of stay and C. diff infections

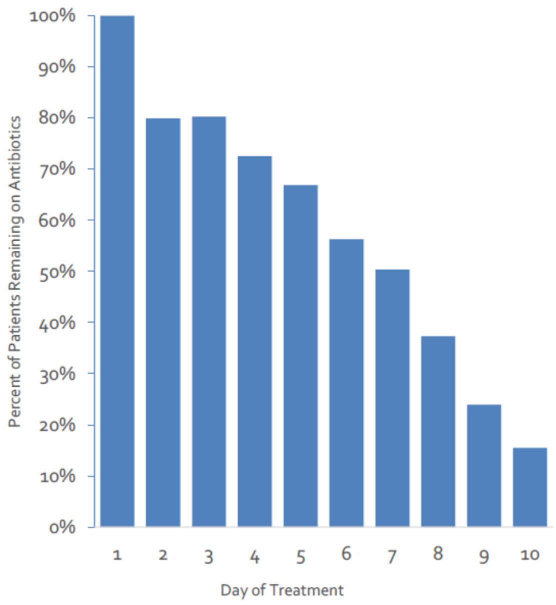

Source: Petty LA, et al. *OFID* 2020

© 2023 Vaughn VA et al. *JAMA Intern Med.*

HMS Pillar Three:
Pay for Performance Metrics

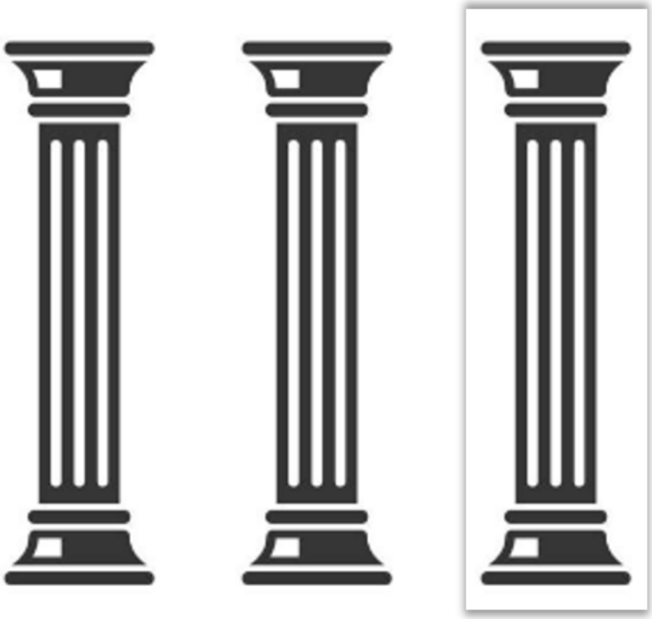

© 2023 Vaughn VA et al. *JAMA Intern Med.*

Michigan Hospital Medicine Safety (HMS) Collaborative
Performance Index

Each year, HMS establishes a series of metrics to determine hospital's progress and participation in the collaborative. To measure each participating hospital's performance, HMS leadership creates a performance index in which each hospital is scored on yearly. This performance index is used to determine participating hospitals' overall pay for performance payment. Hospitals participating in HMS may also participate in other clinical quality initiatives (CQIs) sponsored by Blue Cross Blue Shield of Michigan (BCBSM); each CQI is required by BCBSM to create a performance index. Each hospital's yearly pay for performance payment factors in the scores received across all CQIs the hospital participates in. Each CQI, including HMS modifies their performance index yearly to account for initiative changes and hospital improvement over time. The HMS performance index is approved by both the HMS DDP (Data, Design and Publications Committee), which serves as our technical expert and stakeholder panel and BCBSM. The performance index totals 100 points. Hospitals are assessed on an average of 10 performance measures during a given performance year which occurs during a calendar year.

Core Tenants of the Performance Index

Participation
Being highly engaged in HMS is critical in assessing and improving in the collaborative initiatives. As such, participation at HMS meetings and submission of timely and accurate data are key factors in which hospitals are assessed. During the performance year, 30 points out of 100 are dedicated to participation and timeliness of data collection.

Setting Hospital-Specific Performance Measures
Each hospital is assessed on their performance related to key quality measures determined by HMS. Typically, the measure consists of three thresholds for point determination; full points, partial points, and no points; these thresholds are evaluated and updated on an annual basis using a methodology to determine the scores associated with each threshold. For each individual measure, the collective scores for all participating hospitals are analyzed to determine percentiles of performance, including the top 25%, top 33% and top 50% of hospitals. When setting a full point threshold, we start with the score that reflects the top 25% of hospitals, as the goal is to continue to drive increased improvement from year to year. For methodologic rigor, the percentage of patients within the score that reflects the top 25% of hospitals must be greater than 10%. Reference here. Next, we assess whether there are unintended consequences for setting the measure at the given threshold. Similarly, we determine if we are comfortable with the true improvement opportunity. Specifically, if we believe the gaps in performance are due to limitations in data collection vs. a true opportunity for improvement. To assist in assessing these key factors, hospitals review their fallout cases regularly and communicate potential issues with data collection or clinical scenarios. All of these factors are considered when setting thresholds for a given measure. If, after this assessment, the top 25% fails one of the above factors, the same process is followed at both the top 33% and 50% of hospitals.

Collaborative Wide Measures
BCBSM requires that each CQI maintain a collaborative wide measure. A collaborative measure is calculated by taking an average score of all the participating hospitals during the fourth quarter of every year. If the collaborative meets the pre-determined point threshold, then all of the hospitals achieve the points. If the collaborative does not meet the pre-determined point threshold then, none of the hospitals achieve the points, including those who individually achieved the threshold.

ASB performance metrics – 2018 to 2021

Since 2018, ASB has been part of the performance index. Below are the performance index scorecards for each year with associated notes. In all years, antibiotic treatment was assessed on day 2 after the positive urine culture (i.e., patient may receive one day of antibiotic therapy and still meet the metric)—this decision was made given that HMS is primarily a hospital medicine collaborative and initial doses of antibiotics are often given in the emergency department.

© 2023 Vaughn VA et al. *JAMA Intern Med.*

2018 Michigan Hospital Medicine Safety Consortium Collaborative Quality Initiative Performance Index Scorecard

2018 Michigan Hospital Medicine Safety Consortium Collaborative Quality Initiative Performance Index Scorecard	
Measure Description	Points
≤40% of cases treated for a UTI are actually ASB **AND** ≤80% of patients with ASB receive antibiotics	15 (full)
≤40% of cases treated for a UTI are actually ASB **OR** ≤80% of patients with ASB receive antibiotics	10 (partial)
>40% of cases treated for a UTI are actually ASB **AND** >80% of patients with ASB receive antibiotics	0 (none)

2019 Michigan Hospital Medicine Safety Consortium Collaborative Quality Initiative Performance Index Scorecard

2019 Michigan Hospital Medicine Safety Consortium Collaborative Quality Initiative Performance Index Scorecard	
Measure Description	Points
≤35% of cases treated for a UTI are actually ASB **AND** ≤70% of patients with ASB receive antibiotics	15 (full)
≤35% of cases treated for a UTI are actually ASB **OR** ≤70% of patients with ASB receive antibiotics	10 (partial)
>35% of cases treated for a UTI are actually ASB **AND** >70% of patients with ASB receive antibiotics	0 (none)

2020 Michigan Hospital Medicine Safety Consortium Collaborative Quality Initiative Performance Index Scorecard

Hospitals did not have pay-for-performance metrics in 2020 due to the COVID-19 pandemic.

2021 Michigan Hospital Medicine Safety Consortium Collaborative Quality Initiative Performance Index Scorecard

2021 Michigan Hospital Medicine Safety Consortium Collaborative Quality Initiative Performance Index Scorecard	
Measure Description	Points
≤15% of cases treated for a UTI are actually ASB	10 (full)
16-25% of cases treated for a UTI are actually ASB	5 (partial)
>25% of cases treated for a UTI are actually ASB	0 (none)

2022 Michigan Hospital Medicine Safety Consortium Collaborative Quality Initiative Performance Index Scorecard

2022 Michigan Hospital Medicine Safety Consortium Collaborative Quality Initiative Performance Index Scorecard	
Measure Description	Points
≤12% of cases treated for a UTI are actually ASB **OR** ≥33% relative decrease in the number of cases treated for a UTI are actually ASB during the current performance year	10 (full)
13-22% of cases treated for a UTI are actually ASB **OR** 20- 32% relative decrease in the number of cases treated for a UTI are actually ASB during the current performance year	5 (partial)
>22% of cases treated for a UTI are actually ASB **AND** <20% relative decrease in the number of cases treated for a UTI are actually ASB during the current performance year	0 (none)

*relative decrease targets were added to account for hospitals with very high treatment rates who made remarkable improvement over the course of the year

Abbreviations: ASB, asymptomatic bacteriuria; UTI, urinary tract infection

© 2023 Vaughn VA et al. *JAMA Intern Med.*

Michigan Hospital Medicine Safety (HMS) Collaborative
Data Quality Assurance Processes

Details on the HMS Databases

All of the HMS databases use both Drupal and LimeSurvey software for data collection/abstraction, which are maintained by the HMS Coordinating Center and our database administrative team. For data reporting, HMS uses Business Objects software to allow hospitals participating in HMS to access their updated data on a daily basis. For resource gathering and abstraction queries, HMS utilizes a link to a Zendesk guide, which allows abstractors to submit questions regarding data abstraction/reporting, obtain updated data definitions and access resources and quality improvement tools.

Upgrades

Upgrades to the HMS databases occur at least once per year. Upgrades may occur more frequently, depending on updates made to the project throughout the year and changing data needs for quality improvement projects. During an upgrade, the HMS database undergoing updates is taken offline and is unavailable for data abstraction. Upgrades may occur if we have spelling or grammatical errors to fix, selections to add or remove, questions to add or remove, branching questions to add or remove, and/or functionalities to improve or update. After completion of an upgrade, data entered prior to the upgrade is archived and restored in the database. If new questions are added, the abstractors are not expected to return to previously entered cases to enter new data fields as case entry can span several years. The HMS Coordinating Center keeps track of all updates using a ticketing system and the data analytics and statistician team are made aware of all updates.

Data Validation: Audits

Audits are conducted to ensure that the data is being collected consistently across all participating hospitals. The goal is to identify issues with the abstraction process so that they can be appropriately addressed via education and/or changes to the data entry system. Each HMS-participating hospital is audited by a trained member of the Coordinating Center at least once per year. On average ~50 audits have been conducted per year since the launch of HMS in 2011. This number increases each year as new hospitals join the collaborative. It is the expectation that each audited site will attain a 95% or greater rate of accuracy to receive full points on the HMS Performance Index. To determine the audit score, the auditor calculates a score for each individual case based on the average number of audit fields as noted below (see Medical Record Review). Then using the individual scores for each case, an overall audit score is calculated by averaging all of the audit cases combined. If a site receives a score of less than 95% on an audit, every attempt will be made to re-audit that site in the same year.

The audit consists of four parts: medical record review, review of eligibility lists, review of inclusion/exclusion criteria and practices, and post-audit follow up.

<u>Medical Record Review:</u>
The primary focus of the audit is a medical record review of pre-selected cases by one to three HMS auditors. For each initiative, key complication cases are required to be audited to ensure accurate outcome measures for reporting purposes. For our antimicrobial use initiative, all cases with *C. difficile* infection undergo a required audit.

© 2023 Vaughn VA et al. *JAMA Intern Med.*

Prior to the audit, the primary auditor queries the data analytics team to obtain the list of required complication cases that are due for audit and a random sample of additional non-complication cases. On average 7 to 10 cases are audited if one auditor is present. If a site has a large number of unaudited complication cases, a second or third auditor will join to complete additional cases. The list of cases is distributed to the abstractor 1 to 2 weeks in advance of the audit. Prior to sending the list of audited cases, the abstractor is locked from making updates to previously completed cases. Upon the on-site audit, the auditor(s) independently reviews the medical documentation for each case from the hospital's Electronic Medical Record (EMR) and compares it to what was entered into the HMS database. At the end of the audit day, the auditor's case findings and discrepancies between the EMR and the information entered into the HMS databases (if applicable) will be reviewed in detail with the abstractor. At the resolution of the audit, these discrepancies (if validated as incorrect by both the abstractor and auditor), are corrected in the database by the abstractor to ensure case accuracy. The auditor will also provide additional education, as needed, as issues are identified. If during the medical record review, a completed eligible case is determined by the auditor to be ineligible, a score of 90% is assigned to the case and added to the overall average score.

Eligibility List Review:

The second item reviewed during an audit is the eligibility/discharge lists and coding at the site being audited. Prior to the audit, the abstractor connects with their hospital's information technology (IT) group for the coding used to generate their eligibility/discharge lists for each project PICC/Midline (one list for both) and Antimicrobial (separate lists for Pneumonia and Positive Urine Culture). This coding is reviewed by the auditor and feedback is provided regarding updates that need to be made to the coding, if applicable.

Inclusion/Exclusion Criteria Review:

The final item reviewed during an audit is inclusion/exclusion criteria. The purpose of this review is to ensure that the abstractor understands the inclusion/exclusion criteria for each project and is applying those criteria appropriately when reviewing cases. At least one case for each project deemed ineligible by the abstractor is randomly selected and reviewed with the auditor(s). Once a case is identified, the abstractor shows the auditor(s), in the medical record, the reason the case was excluded from abstraction. If a case was deemed ineligible by the abstractor, but was determined through this review that it was actually eligible for abstraction, another case from the same project will be reviewed until a legitimate ineligible case is found. If the abstractor has incorrectly identified a case as ineligible, the auditor(s) will provide additional on-site education about eligibility criteria.

Post-Audit Follow Up:

After the audit has concluded, the primary auditor composes a summary of the findings, including specific areas to update in the HMS databases, education provided to the site during the audit, and a summary of any findings from the eligibility/discharge list review. The final audit summary is provided to the site within two to three weeks of completion of the audit. This summary will be sent to the site's abstractor(s), quality administrator, and physician champion. The summary will include a percentage score for the audit, which is calculated based on the average of the scores for all cases reviewed. Upon receiving the final audit summary, the abstractor(s) has three months from the date of receipt to make all updates in the HMS database noted in the final report. The final audit score is then factored into the

© 2023 Vaughn VA et al. *JAMA Intern Med.*

site's performance index scorecard for the given year. During a typical year, 5% of the performance index is associated with the audit score(s) completed during the performance year.

Data Validation: Data Checker

Each HMS database has a robust data checker that can identify in real time errors in abstraction that have occurred on a case-by-case basis. Abstractors are trained to run a data checker on each case before submitting it to the database so that any data errors are identified at the time of the initial abstraction and can be corrected prior to submission. Additionally, a live daily report is available, which provides a culmination of all data errors on all cases entered into the database that an abstractor is able to see in order to correct potential discrepancies in data abstraction.

Data Validation: Global Data Checks

The HMS Coordinating Center conducts global data checks on an ad hoc basis during the data analysis process to identify any issues that might occur across the entire database that may not be included in the data checker. These global data checks are typically run when we identify an error as part of another process, such as coding a report and realizing something does not pull into the report as expected. The HMS Coordinating Center will do data queries to sites throughout the year with prompts to analyze their data in accordance with the medical record if we notice discrepancies outside of the data checker elements.

Data Validation: Site Specific

Each hospital receives site specific data reports via a printed version quarterly and daily within the database/registry. Included in these reports are the sites overall score for each measure by quarter and a detailed list of cases that have been identified as opportunities for improvement (i.e., fall-outs). Each hospital is encouraged to review these fall-outs with their local team to perform audit and feedback, identify trends, and assist with overall quality improvement. Occasionally, during this review the local team will identify a potential issue with how the fall-out was determined based on the clinical scenario. In some instances, the case is reviewed and justification for the coding/calculation is reinforced to the local site. In other instances, modifications to the code and/or additional modifications to the data registry questions are required. Typically, the latter is more common at the initial launch of a new measure. For more longstanding measures, modifications are rare.

© 2023 Vaughn VA et al. *JAMA Intern Med.*

Michigan Hospital Medicine Safety (HMS) Collaborative
Definitions for Hospital Characteristics

Antibiotic stewardship team characteristics

- Self-reported data from a November 2019 survey completed by the data abstractor and physician champion of each hospital.
- Survey question as follows:
  - Please select all that applies as it relates to the Director(s)/Leader(s) of your hospital's Antimicrobial Stewardship Program:
    - Infectious Diseases (ID) Physician
    - Physician (Non ID)
    - Infectious Diseases (ID) Pharmacist
    - Pharmacist (non-ID)
    - Other

Academic hospital

- Data obtained from American Hospital Directory, Inc.'s *Individual Hospital Statistics for Michigan.* Retrieved 4/29/2022 from https://www.ahd.com/states/hospital_MI.html

Rural/Urban

- Rurality of individual hospitals was defined using the USDA 2013 Rural-Urban Continuum Codes (RUCC). Retrieved 7/18/2022 from https://www.ers.usda.gov/data-products/rural-urban-continuum-codes.aspx
- The RUCC score ranges from one (least rural) to nine (most rural). Generally, a RUCC score of ≥4 indicates a rural hospital and a score of ≥7 is considered extremely rural.

Profit status

- Data obtained from AHA's Data Hub. Retrieved 4/28/2021 from https://guide.prod.iam.aha.org/guide/searchResults

Bed Size

- Data obtained from 2020 Michigan Certificate of Need Annual Survey, Basic Total Licensed Beds Utilization Statistics. Retrieved 6/21/2022 from https://www.michigan.gov/mdhhs/-/media/Project/Websites/mdhhs/Doing-Business-with-MDHHS/Health-Care-Providers/Certificate-of-Need/CON-Eval/Survey-Reports/2020/Beds/Report-010-Hospital-Beds-by-HSA.pdf?rev=16e5fcc69b6d4a03b17813f6028a34c3&hash=249DA44852A4FC41F010A3EA9D196034

Systemness

- Refers to whether a hospital belongs to a larger state or national healthcare system or is an independent hospital.
- Self-reported data from a November 2019 survey completed by the data abstractor and physician champion of each hospital.

© 2023 Vaughn VA et al. *JAMA Intern Med.*

Michigan Hospital Medicine Safety (HMS) Collaborative
Definition of sex, race, ethnicity

Gender

Instructions: Review the medical record to determine the gender of the patient.
This is a required field and the form cannot be submitted without an entry in this field.
Select one of the following:

- *"Male"* if the patient is categorized as a man in the medical record.
- *"Female"* if the patient is categorized as a woman in the medical record.
- *"Unknown"* if the patient's gender is unknown.

Ethnicity

Instructions: Review the medical record to determine the patient's ethnicity.
Select one of the following:

- *"Hispanic or Latino"* if patient demographic information indicates patient is of Hispanic descent. The US Census Bureau states that "People who identify their origin as Spanish, Hispanic, or Latino may be of any race."
- *"Non-Hispanic or Latino"* if patient demographic information indicates patient is not of Hispanic descent.
- "*Unknown*" if ethnicity is not reported in the medical record.

Race

Instructions: Review the medical record to determine the patient's race.
Select one of the following:

- *"American Indian or Alaskan Native"* if patient demographic information indicates patient is Native American, American Indian, or Alaska Native.
- *"Arab and Chaldean Ancestries"* if the patient demographic information indicate patient is of Arab or Chaldean Ancestries.
- *"Asian"* if patient demographic information indicates Asian.
- *"Black or African American"* if patient demographic information indicates patient is black or African American.
- *"Native Hawaiian or Pacific Islander"* if patient demographic information indicates patient is Native Hawaiian or Pacific Islander.
- *"White or Caucasian"* if patient demographic information indicates patient is white or Caucasian.
- *"Other"* if patient demographic information indicates the patient is a race other than what is listed above.
- *"Unknown"* if patient's race is not indicated in the medical record.

© 2023 Vaughn VA et al. *JAMA Intern Med.*

Antibiotic and Diagnostic Stewardship Strategies

Hospital antibiotic and diagnostic stewardship activities related to ASB were obtained via hospital surveys in 2019. Surveys were administered electronically using Qualtrics XM and emailed to all HMS hospitals on 11/2019 (completed by 12/2019). The data abstractor (typically a nurse in quality) at each hospital was responsible for working with local individuals (e.g., antibiotic stewardship leaders) to ensure survey accuracy and completion. Of the 46 study hospitals, only 40 received the 2019 survey; of those, 97.5% (39/40) responded. An additional 3 hospitals who did not receive the 2019 survey answered a similar November 2018 survey when antibiotic stewardship, but not diagnostic stewardship data, were collected.

HMS Quality Improvement Survey - Fall 2019

Please answer the following questions based on the activity at your hospital over the last six months (unless otherwise specified). The survey is due 11/27/2019.

1. Hospital Name

2. Does your hospital have an antimicrobial stewardship team?

    - Yes
    - No

3. On January 1, 2017 the Joint Commission launched a new standard for hospitals, critical access hospitals and nursing care centers that addresses antimicrobial stewardship. Since January 1, 2017, have stewardship resources increased at your hospital (i.e., additional FTE support, etc.)?

    - Yes
    - No

4. You stated that since January 1, 2017 stewardship resources have increased at your hospital (i.e., additional FTE support). When did this occur?

5. You stated that since January 1, 2017 stewardship resources have increased at your hospital. Please describe the additional resources (i.e., physician FTE, pharmacist, FTE, IT support, etc.):

6. Does your hospital have a policy that requires prescribers to document the following in the daily progress notes and/or discharge summary (Check all that Apply)?

    - Dose
    - Intended Duration of Use
    - Indication
    - None of the above

© 2023 Vaughn VA et al. *JAMA Intern Med.*

7. Does your hospital have an institutional treatment guideline for UTI?

   - Yes and it is a new guideline or has been updated in the past 12 months
   - Yes and it has NOT been updated in the past 12 months
   - No
   - Currently being developed

8. Does your hospital's UTI guideline provide indications for obtaining a urine culture?

   - Yes
   - No

9. Does your hospital's UTI guideline provide recommendations for not treating asymptomatic bacteriuria (ASB)?

   - Yes
   - No

10. Does your hospital's UTI guideline provide antibiotic regimens that are concordant with HMS recommendations?

    - Yes
    - No

11. Does your hospital's UTI guideline recommend fluoroquinolones as a first line agent for cystitis (uncomplicated or complicated)?

    - Yes
    - No

© 2023 Vaughn VA et al. *JAMA Intern Med.*

12. What interventions to reduce inappropriate use of urine cultures (e.g., diagnostic stewardship) has your hospital started?

- Removal/change in urine culture testing from preoperative order sets
- Removal/change of urine culture testing from ED order sets
- Removal/change of urine culture testing from admission order sets
- Removal of urine culture testing from other order sets
- Added reflex testing (urinalysis cutoff to urine cultures)
- Removed reflex testing (urinalysis cutoff to send urine cultures)
- Hiding urine culture results in some settings
- Requiring physician order to run urine cultures in ED
- Other two-step urine culture initiative to reduce urine cultures in ED
- Framing urine culture results in test results (e.g., adding language about asymptomatic bacteriuria)
- Rejection of some urine cultures (e.g., based on squamous cells)
- Other

13. Does your stewardship program perform audit and feedback on patients with any of the following? (Select all that apply)

- Pneumonia (CAP or HCAP)
- Asymptomatic Bacteriuria (ASB)
- Urinary Tract Infection (UTI)
- None of the above

14. Does your computerized physician order entry (CPOE) contain order sets specific for patients with UTI which incorporates your facility-specific treatment recommendations?

- Yes
- No

15. Does your computerized physician order entry (CPOE) contain decision support to discourage the attainment of urine culture in asymptomatic patients?

- Yes
- No

© 2023 Vaughn VA et al. *JAMA Intern Med.*

16. Does your hospital have a formal procedure/policy for reviewing the appropriateness of all antibiotics after the initial orders (e.g., a "timeout" 48-72 hours after starting antibiotics)?

- Yes
- No

17. Are any of the following Fluoroquinolones non-formulary?

- Ciprofloxacin (Cipro, Ciproxin, Ciprobay)
- Delafloxacin (Baxdela)
- Levofloxacin (Levaquin, Quixin)
- Moxifloxacin (Avelox)
- Not Applicable

18. Does your stewardship program provide education to clinicians and other staff on improving antibiotic prescribing for patients with UTI and Asymptomatic Bacteriuria (ASB)?

- Yes
- No

© 2023 Vaughn VA et al. *JAMA Intern Med.*

Revised Standards for Quality Improvement Reporting Excellence (SQUIRE 2.0)
September 15, 2015

Text Section and Item Name	Section or Item Description	
**Notes to authors**	• The SQUIRE guidelines provide a framework for reporting new knowledge about how to improve healthcare<br><br>• The SQUIRE guidelines are intended for reports that describe system level work to improve the quality, safety, and value of healthcare, and used methods to establish that observed outcomes were due to the intervention(s).<br><br>• A range of approaches exists for improving healthcare. SQUIRE may be adapted for reporting any of these.<br><br>• Authors should consider every SQUIRE item, but it may be inappropriate or unnecessary to include every SQUIRE element in a particular manuscript.<br><br>• The SQUIRE Glossary contains definitions of many of the key words in SQUIRE.<br><br>• The Explanation and Elaboration document provides specific examples of well-written SQUIRE items, and an in-depth explanation of each item.<br><br>• Please cite SQUIRE when it is used to write a manuscript.	
**Title and Abstract**		
**1. Title**	Indicate that the manuscript concerns an initiative to improve healthcare (broadly defined to include the quality, safety, effectiveness, patient-centeredness, timeliness, cost, efficiency, and equity of healthcare)	1
**2. Abstract**	a. Provide adequate information to aid in searching and indexing<br>b. Summarize all key information from various sections of the text using the abstract format of the intended publication or a structured summary such as: background, local problem, methods, interventions, results, conclusions	5

© 2023 Vaughn VA et al. *JAMA Intern Med.*

Introduction	*Why did you start?*	
**3. Problem Description**	Nature and significance of the local problem	7
**4. Available knowledge**	Summary of what is currently known about the problem, including relevant previous studies	7
**5. Rationale**	Informal or formal frameworks, models, concepts, and/or theories used to explain the problem, any reasons or assumptions that were used to develop the intervention(s), and reasons why the intervention(s) was expected to work	7
**6. Specific aims**	Purpose of the project and of this report	7
**Methods**	*What did you do?*	
**7. Context**	Contextual elements considered important at the outset of introducing the intervention(s)	8
**8. Intervention(s)**	a. Description of the intervention(s) in sufficient detail that others could reproduce it<br>b. Specifics of the team involved in the work	9 - 10
**9. Study of the Intervention(s)**	a. Approach chosen for assessing the impact of the intervention(s)<br>b. Approach used to establish whether the observed outcomes were due to the intervention(s)	9 - 10
**10. Measures**	a. Measures chosen for studying processes and outcomes of the intervention(s), including rationale for choosing them, their operational definitions, and their validity and reliability<br>b. Description of the approach to the ongoing assessment of contextual elements that contributed to the success, failure, efficiency, and cost<br>c. Methods employed for assessing completeness and accuracy of data	9 - 10
**11. Analysis**	a. Qualitative and quantitative methods used to draw inferences from the data<br>b. Methods for understanding variation within the data, including the effects of time as a variable	11 - 12
**12. Ethical Considerations**	Ethical aspects of implementing and studying the intervention(s) and how they were addressed, including, but not limited to, formal ethics review and potential conflict(s) of interest	12

© 2023 Vaughn VA et al. *JAMA Intern Med.*

Results	*What did you find?*	
**13. Results**	a. Initial steps of the intervention(s) and their evolution over time (*e.g.*, time-line diagram, flow chart, or table), including modifications made to the intervention during the project<br>b. Details of the process measures and outcome<br>c. Contextual elements that interacted with the intervention(s)<br>d. Observed associations between outcomes, interventions, and relevant contextual elements<br>e. Unintended consequences such as unexpected benefits, problems, failures, or costs associated with the intervention(s).<br>f. Details about missing data	12 - 14
**Discussion**	*What does it mean?*	
**14. Summary**	a. Key findings, including relevance to the rationale and specific aims<br>b. Particular strengths of the project	14 - 15
**15. Interpretation**	a. Nature of the association between the intervention(s) and the outcomes<br>b. Comparison of results with findings from other publications<br>c. Impact of the project on people and systems<br>d. Reasons for any differences between observed and anticipated outcomes, including the influence of context<br>e. Costs and strategic trade-offs, including opportunity costs	15
**16. Limitations**	a. Limits to the generalizability of the work<br>b. Factors that might have limited internal validity such as confounding, bias, or imprecision in the design, methods, measurement, or analysis<br>c. Efforts made to minimize and adjust for limitations	15 - 16
**17. Conclusions**	a. Usefulness of the work<br>b. Sustainability<br>c. Potential for spread to other contexts<br>d. Implications for practice and for further study in the field<br>e. Suggested next steps	16
**Other information**		
**18. Funding**	Sources of funding that supported this work. Role, if any, of the funding organization in the design, implementation, interpretation, and reporting	17

© 2023 Vaughn VA et al. *JAMA Intern Med.*

eFigure 2. Study Flow Diagram

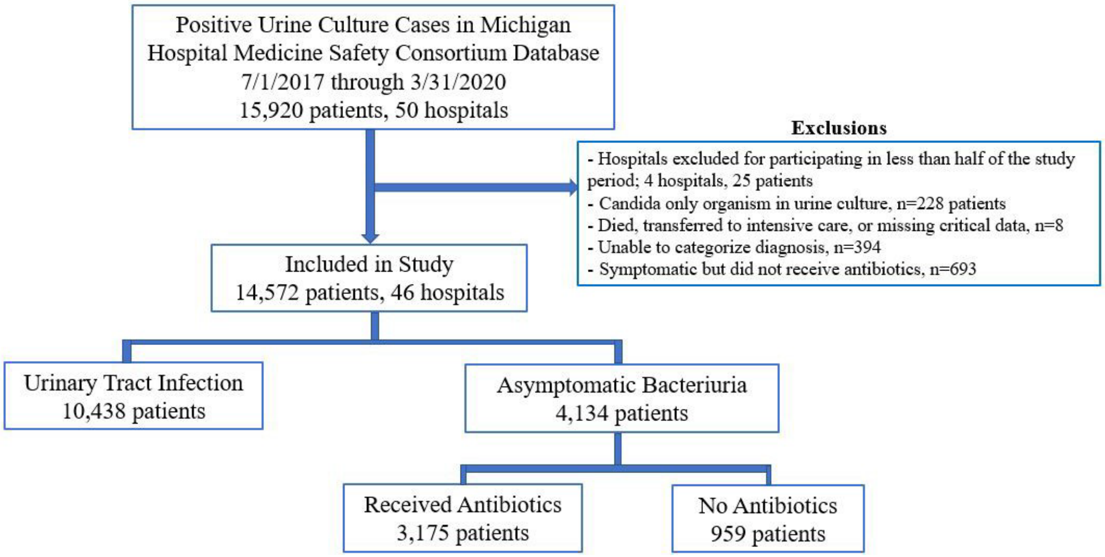

© 2023 Vaughn VA et al. *JAMA Intern Med.*

eTable 1. Antibiotic Treatment in Patients With UTI or ASB Who Were Treated With Antibiotics
N=13,613 patients across 46 hospitals

Characteristic	UTI treated with Antibiotics N=10,438	ASB treated with Antibiotics N=3,175
Duration of Therapy		
Days; Median (IQR)	8 (5-11)	6 (4-8)
≥3 Days; n (%)	9470 (93.3%)	2675 (84.3%)
Empiric Antibiotics; n (%)		
Ceftriaxone	7542 (72.3%)	2233 (70.3%)
Fluoroquinolone^	1037 (9.9%)	336 (10.6%)
Cephalosporin (1st or 2nd generation)	937 (9.0%)	242 (7.6%)
Piperacillin/tazobactam	473 (4.5%)	55 (1.7%)
Trimethoprim/sulfamethoxazole	173 (1.7%)	81 (2.6%)
Fosfomycin	118 (1.1%)	59 (1.9%)
Nitrofurantoin	130 (1.2%)	64 (2.0%)
Ampicillin/sulbactam	62 (0.6%)	11 (0.3%)
Other	1409 (13.5%)	252 (7.9%)
Missing antibiotic name	114 (1.1%)	77 (2.4%)
Antibiotics at Discharge; n (%)	7405 (70.9%)	1799 (56.7%)
Cephalosporin (1st , 2nd, or 3rd generation)	2813 (38.0%)	693 (38.5%)
Fluoroquinolone^	2364 (31.9%)	546 (30.4%)
Trimethoprim/sulfamethoxazole	832 (11.2%)	181 (10.1%)
Nitrofurantoin	393 (5.3%)	120 (6.7%)
Fosfomycin	76 (1.0%)	19 (1.1%)
Other	1118 (15.1%)	264 (14.7%)
Ordering Provider; n (%)		
Ordered urine culture*		
EM provider	6632/8510 (77.9%)	1686/2447 (68.9%)
Other	1878/8510 (22.1%)	761/2447 (31.1%)
Ordered antibiotic*		
EM provider	5981/8501 (70.4%)	1349/2410 (56.0%)
Other	2520/8501 (29.6%)	1061/2410 (44.0%)

^ Includes ciprofloxacin, levofloxacin, or moxifloxacin.
* Only a subset of patients had these data collected.

Abbreviations: ASB: asymptomatic bacteriuria; UTI: urinary tract infection; IQR: inter-quartile range; EM: emergency medicine

© 2023 Vaughn VA et al. *JAMA Intern Med.*

eTable 2A. Association of Hospital Characteristics With Baseline Rate and Change in Diagnostic Stewardship Metric (Percentage of Hospitalized Patients With a Positive Urine Culture Who Had ASB)
N=46 hospitals with 13,613 Patients

Hospital Characteristic	N (%) of hospitals, N=46 Hospitals	Interaction Effect with Baseline Treatment OR or IRR (95% CI)	Interaction Effect with Change Over Time OR or IRR (95% CI)
Antibiotic Stewardship Team Leader[a]			
ID Physician and ID Pharmacist	18 (46.2%)	REF	REF
ID Physician or ID Pharmacist	16 (41.0%)	1.00 (0.73-1.38)	1.03 (0.99-1.07)
Non-ID trained	5 (12.8%)	0.94 (0.55-1.58)	1.06 (0.99-1.13)
Academic Hospital[b]	38 (82.6%)	0.89 (0.61-1.30)	1.00 (0.94-1.06)
RUCC Score[c]; median (IQR)	2 (1-3)	1.05 (0.97-1.13)	1.01 (0.997-1.02)
1-3 (non-rural)	37 (80.4%)	REF	REF
4-9 (rural)	5 (10.9%)	0.85 (0.51-1.43)	1.08 (0.98-1.19)
7-9 (very rural)	4 (8.7%)	1.32 (0.80-2.16)	1.04 (0.97-1.12)
Profit Type[d]			
Non-profit	39 (84.8%)	REF	REF
For profit	5 (10.9%)	0.99 (0.64-1.53)	1.02 (0.97-1.08)
Bed Size[e]; median (IQR)	308 (186-443)	0.97 (0.92-1.03)	1.00 (0.99-1.01)
System[f]			
Independent	4 (8.7%)	0.88 (0.52-1.51)	0.95 (0.88-1.02)
State	19 (41.3%)	0.81 (0.61-1.08)	1.02 (0.99-1.06)
National	23 (50.0%)	REF	REF

eTable 2B. Association of Hospital Characteristics With Baseline Rate and Improvement in Antibiotic Stewardship Metric (Percentage of ASB Patients Who Were Treated with Antibiotics)
N=46 hospitals with 4,134 Patients

Hospital Characteristic	N (%) of hospitals, N=46 Hospitals	Interaction Effect with Baseline Treatment OR or IRR (95% CI)	Interaction Effect with Improvement in Antibiotic Use Over Time OR or IRR (95% CI)
Antibiotic Stewardship Team Leader[a]			
ID Physician and ID Pharmacist	18 (46.2%)	REF	REF
ID Physician or ID Pharmacist	16 (41.0%)	1.28 (0.72-2.27)	1.04 (0.97-1.12)
Non-ID trained	5 (12.8%)	0.75 (0.30-1.89)	1.11 (0.99-1.25)
Academic Hospital[b]	38 (82.6%)	0.94 (0.46-1.91)	1.03 (0.93-1.15)
RUCC Score[c]; median (IQR)	2 (1-3)	0.95 (0.83-1.10)	1.00 (0.98-1.02)
1-3 (non-rural)	37 (80.4%)	REF	REF
4-9 (rural)	5 (10.9%)	1.35 (0.49-3.73)	0.92 (0.75-1.11)
7-9 (very rural)	4 (8.7%)	0.68 (0.28-1.66)	1.06 (0.93-1.20)
Profit Type[d]			
Non-profit	39 (84.8%)	REF	REF
For profit	5 (10.9%)	0.74 (0.34-1.63)	**1.12 (1.01-1.23)***
Bed Size[e]; median (IQR)	308 (186-443)	0.96 (0.86-1.06)	1.00 (0.98-1.01)
System[f]			
Independent	4 (8.7%)	**3.69 (1.14-11.89)***	**0.75 (0.64-0.88)***
State	19 (41.3%)	0.74 (0.44-1.25)	**0.93 (0.88-0.99)***
National	23 (50.0%)	REF	REF
Baseline % of patients treated for UTI who actually had ASB; median (IQR)[g]	26.0% (21.7-31.3)	1.22 (0.98-1.53)	1.00 (0.97-1.03)

© 2023 Vaughn VA et al. *JAMA Intern Med.*

In the models assessing change over time in the percentage of patients who had ASB and the percentage of patients with ASB who received antibiotics, we added potential hospital level explanatory variables (e.g., bed size) to describe how rate of change varied by these factors. The interaction effect with baseline treatment demonstrates which hospital characteristics were associated with baseline rates of ASB (or ASB treatment). The slope's interaction effect demonstrates whether the hospital characteristic is associated with change over time with OR/IRR>1 indicating less of a decrease over time. * Statistically significant value

[a] Data are self-reported from a survey completed by each hospital in November 2019. Data missing for 7 hospitals.
[b] Academic hospital status from the American Medical Association's Fellowship and Residency Electronic Interactive Database Institution Directory.
[c] Rurality of individual hospitals was defined using the RUCC score, ranging from one (least rural) to nine (most rural). Generally, a RUCC score of ≥4 indicates a rural hospital.
[d] Profit status obtained from data.medicare.gov. Excludes 2 governmental hospitals.
[e] Hospital bed size was obtained from the 2015 Michigan Certificate of Need Annual Survey. OR and IRR shown per 100 hospital beds.
[f] Refers to whether hospital belongs to a larger healthcare system. Data obtained from the Michigan Health and Hospital Association Systems Listing (https://www.mha.org/About/Our-Hospitals/Michigan-Hospitals-By-Health-System).

Abbreviations: OR, odds ratio (used for categorical outcomes). IRR, incidence rate ratio (used for continuous outcomes). CI, confidence interval. UTI, urinary tract infection. ASB, asymptomatic bacteriuria. ID, infectious diseases. RUCC, rural-urban continuum codes. IQR, inter-quartile range.

© 2023 Vaughn VA et al. *JAMA Intern Med.*

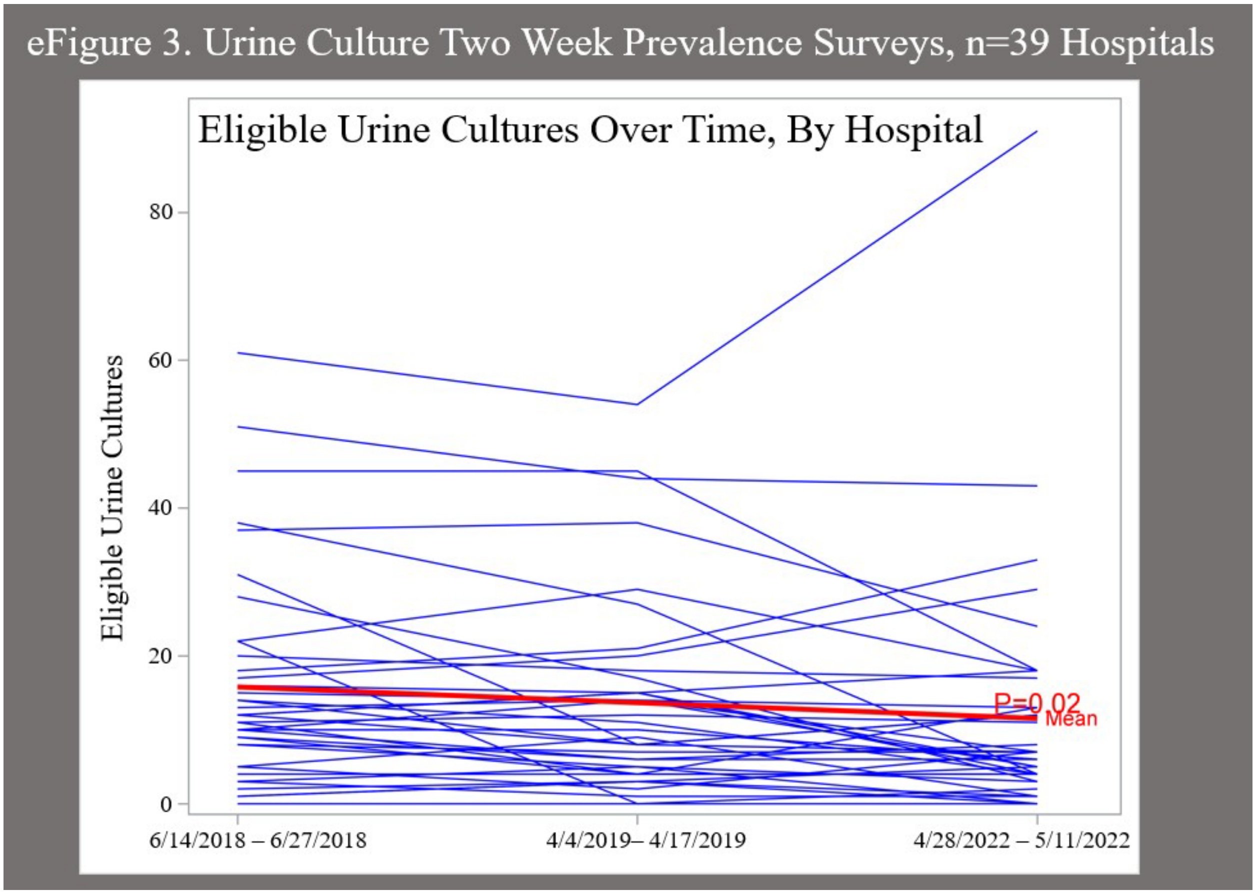

To estimate whether the number of urine cultures decreased over time, HMS conducted three separate two week-long prevalence surveys of urine cultures. During the time periods (6/14/2018 – 6/27/2018; 4/4/2019 – 4/17/2019, and 4/28/2022 – 5/11/2022), we asked data abstractors to collect the following data a) how many total positive urine culture cases were included on your hospitals' patient/eligibility discharge list and b) how many positive urine cultures cases were eligible for abstraction based on HMS eligibility criteria? Results of the total number of patients eligible for abstraction are shown. The red line represents the mean number of eligible urine cultures across all hospitals; blue lines represent individual hospitals. Overall, we found a statistically significant decrease in eligible urine culture cases over time.

© 2023 Vaughn VA et al. *JAMA Intern Med.*

eFigure 4. Distribution of Hospital Bed Size Over Time, n=39 Hospitals

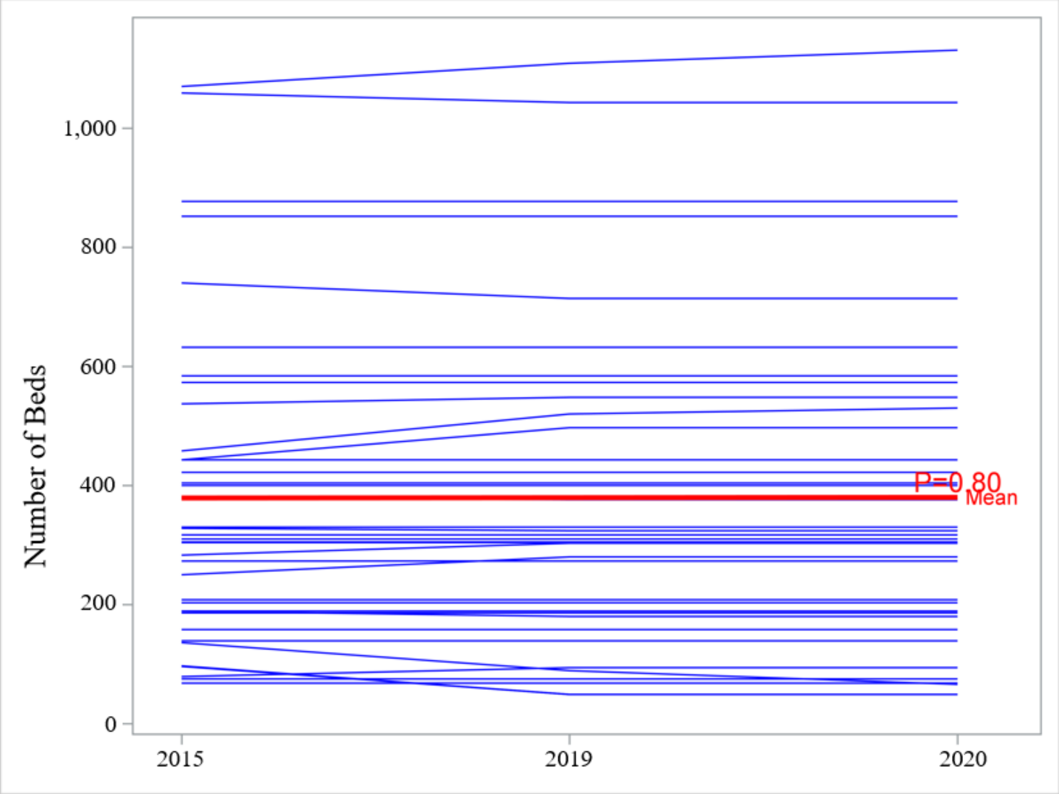

To ensure the decrease in urine cultures was not due to a decrease in bed size, we tracked bed size over time for included hospitals. Bed size data were obtained from the Michigan Certificate of Need Annual Survey, Basic Total Licensed Beds Utilization Statistics in 2015, 2019 and 2020. https://www.michigan.gov/mdhhs/-/media/Project/Websites/mdhhs/Doing-Business-with-MDHHS/Health-Care-Providers/Certificate-of-Need/CON-Eval/Survey-Reports/2020/Beds/Report-010-Hospital-Beds-by-HSA.pdf?rev=16e5fcc69b6d4a03b17813f6028a34c3&hash=249DA44852A4FC41F010A3EA9D196034

The red line represents the mean number of beds across all hospitals; blue lines represent individual hospitals. Overall, we found no statistically significant change in bed size over time.

© 2023 Vaughn VA et al. *JAMA Intern Med.*

CHANGE OVER TIME IN OBJECTIVE SIGNS OR SUBJECTIVE SYMPTOMS IN PATIENTS WITH UTI
n=10,438 PATIENTS

Objective Signs:

- Any Objective Sign (aOR per quarter: 0.99, 95% CI: 0.97, 1.00)
- Hypotension (aOR per quarter: 1.00, 95% CI: 0.99, 1.02)
- Leukocytosis (aOR per quarter: 1.01, 95% CI: 0.99, 1.02)
- SIRS criteria over time (aOR per quarter: 0.99, 95% CI: 0.98, 1.01)
- Fever decreased over time (aOR per quarter 0.98, 95% CI: 0.96, 0.998)

Subjective symptoms:

- **Any subjective symptom (aOR per quarter: 1.03, 95% CI: 1.00, 1.05, p=0.04)**
- Urgency (aOR per quarter 1.01, 95% CI: 0.99 1.04)
- Rigors (aOR per quarter 0.98, 95% CI: 0.95, 1.02)
- Frequency (aOR per quarter 1.01, 95% CI: 0.99, 1.03)
- **Dysuria (aOR per quarter 1.02, 95% CI: 1.01, 1.04)**
- **Suprapubic pain/tenderness (aOR per quarter 1.05, 95% CI: 1.02, 1.08)**
- **Acute hematuria (aOR per quarter 1.03, 95% CI: 1.01, 1.05)**
- Costovertebral/flank pain/tenderness (aOR per quarter1.01, 95% CI: 0.99, 1.03)
- Documentation of pyelonephritis (aOR per quarter 1.02, 95% CI: 0.99, 1.04)
- Altered mental status (aOR per quarter: 1.00, 95% CI: 0.98, 1.02)

To evaluate for possible documentation changes, we assessed for change over time in objective signs (fever, hypotension, leukocytosis, SIRS criteria) vs. subjective symptoms assessed via documentation (urgency, rigors, frequency, dysuria, suprapubic pain/tenderness, acute hematuria, costovertebral/flank pain/tenderness, documentation of pyelonephritis, altered mental status) in patients classified as UTI. Items bolded are statistically significant.

Abbreviations: SIRS, systemic inflammatory response syndrome; UTI, urinary tract infection; aOR, adjusted odds ratio

SIRS: ≥2 of the following: Temperature >38°C or <36°C; Heart rate >90 bpm; Respiratory rate >20 bpm; white blood cell count >12,000/mm³ or <4,000/mm³

© 2023 Vaughn VA et al. *JAMA Intern Med.*
