# Supplementary material for: A Statewide Quality Initiative to Reduce Unnecessary Antibiotic Treatment of Asymptomatic Bacteriuria
Source: JAMA Intern Med. 2023 Jul 10;183(9):933–41. doi: 10.1001/jamainternmed.2023.2749 (PMC10334295; doi:10.1001/jamainternmed.2023.2749)
Supplement: Supplement 2. — Data Sharing Statement [file jamainternmed-e232749-s002.pdf]

Data Sharing Statement

Vaughn. A Statewide Quality Initiative to Reduce Unnecessary Antibiotic Treatment of Asymptomatic Bacteriuria. *JAMA Intern Med*. Published July 10, 2023. doi:10.1001/jamainternmed.2023.2749

Data
Data available: No

Additional Information
Explanation for why data not available: The Michigan Hospital Medicine Safety consortium's data use agreement between all member hospitals does not allow for data sharing without approval.
